# Supplementary figures and images for: Associations of maternal quitting, reducing, and continuing smoking during pregnancy with longitudinal fetal growth: Findings from Mendelian randomization and parental negative control studies
Source: PLoS Med. 2019 Nov 13;16(11):e1002972. doi: 10.1371/journal.pmed.1002972 (PMC6853297; doi:10.1371/journal.pmed.1002972)

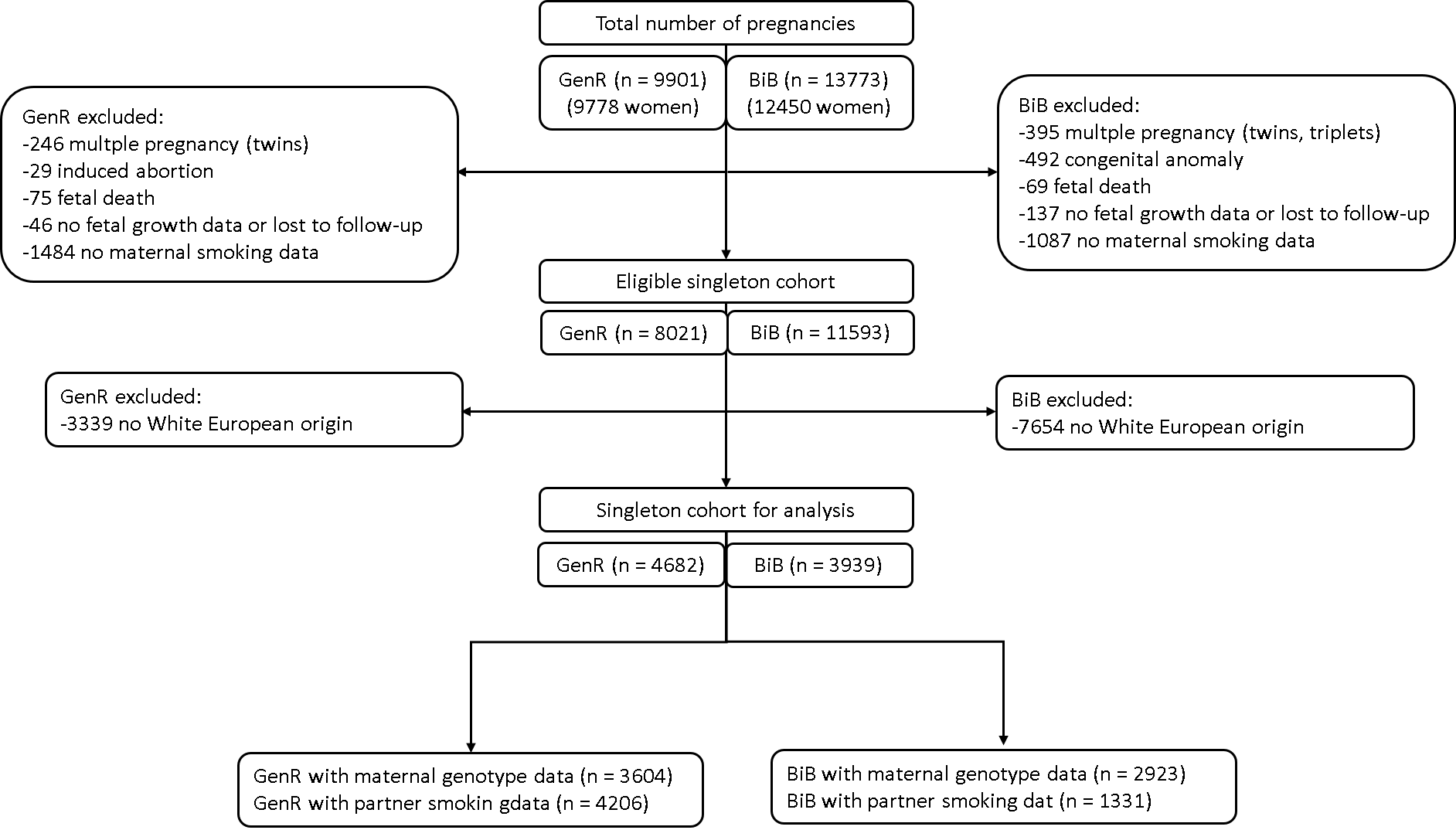

Supplement: S1 Fig — (TIF) [file pmed.1002972.s001.tif]

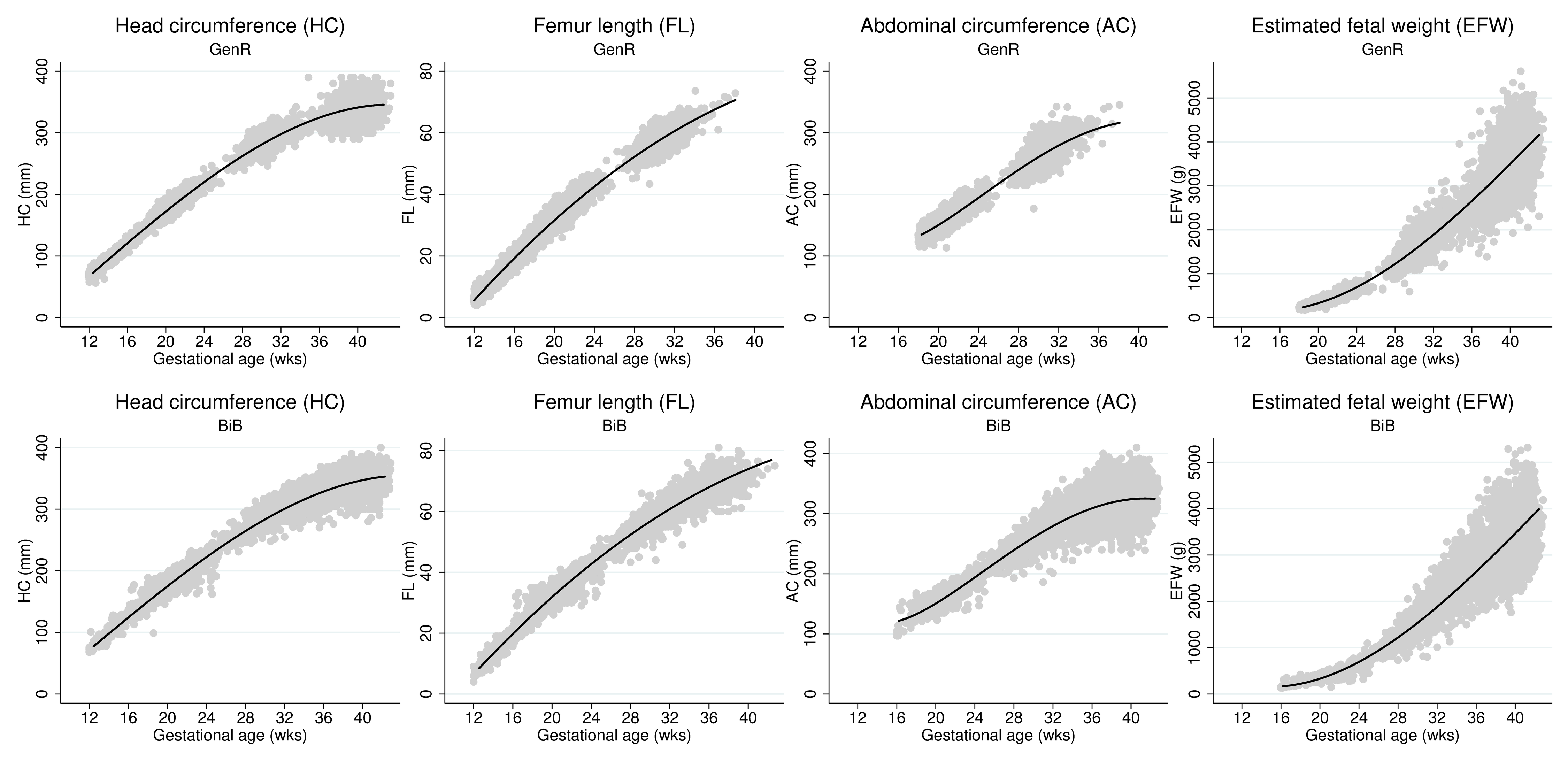

Supplement: S2 Fig — Average growth trajectories of fetal HC, FL, AC, and EFW from 12–16 to 40 weeks’ gestation predicted by best-fitting multilevel fractional polynomial models in GenR and BiB. (TIF) [file pmed.1002972.s002.tif]

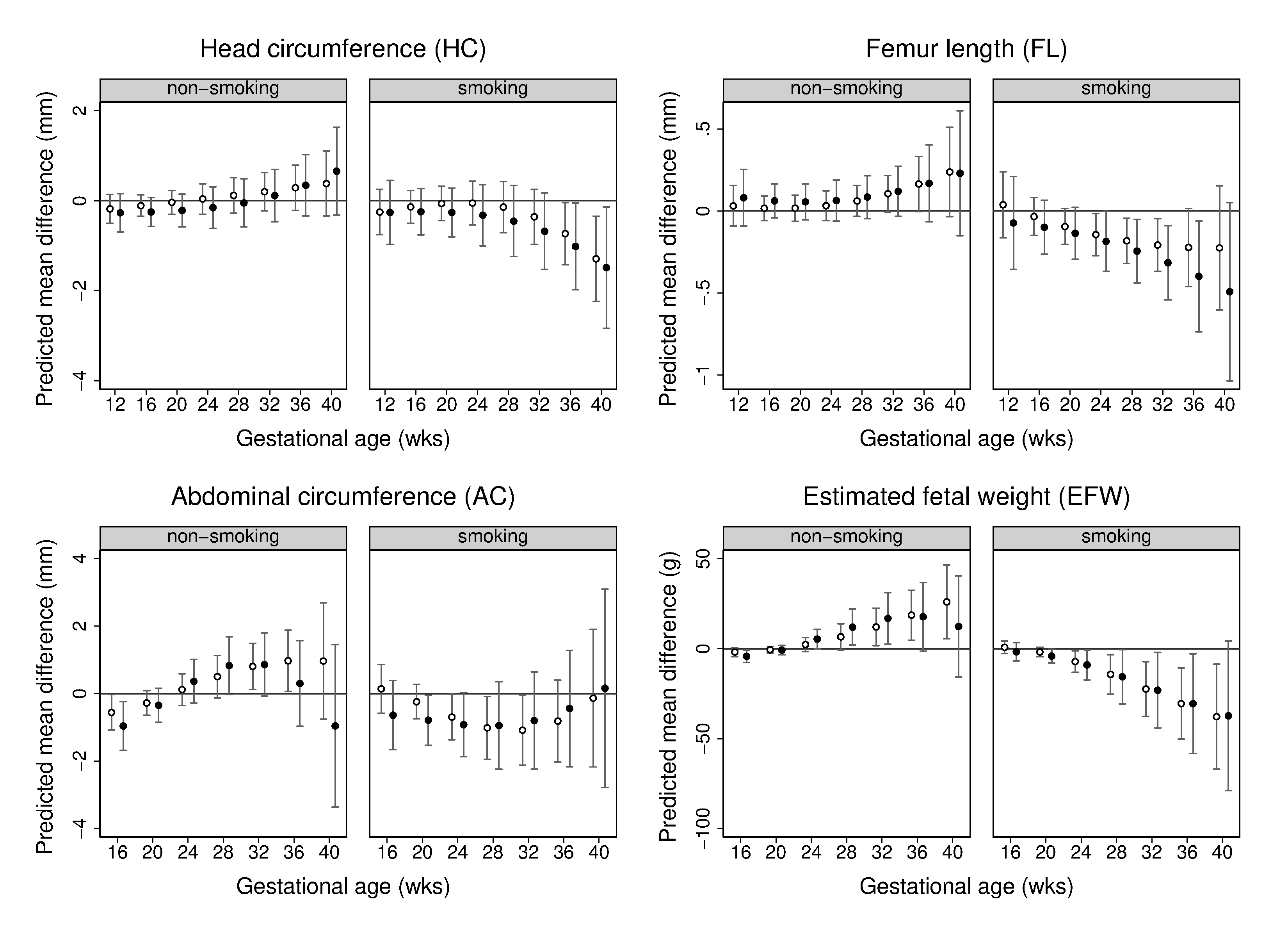

Supplement: S3 Fig — Predicted differences in mean HC (mm), FL (mm), AC (mm), and EFW (g) across gestation per maternal rs1051730 T allele increase in pre-pregnancy smokers and non-smokers. Predicted mean differences (with 95% confidence intervals) in the pooled GenR and BiB cohort by analysis model: model 1 adjusting for cohort (in white) and model 2 adjusting for cohort and fetal rs1051730 genotype (in black). (TIF) [file pmed.1002972.s003.tif]

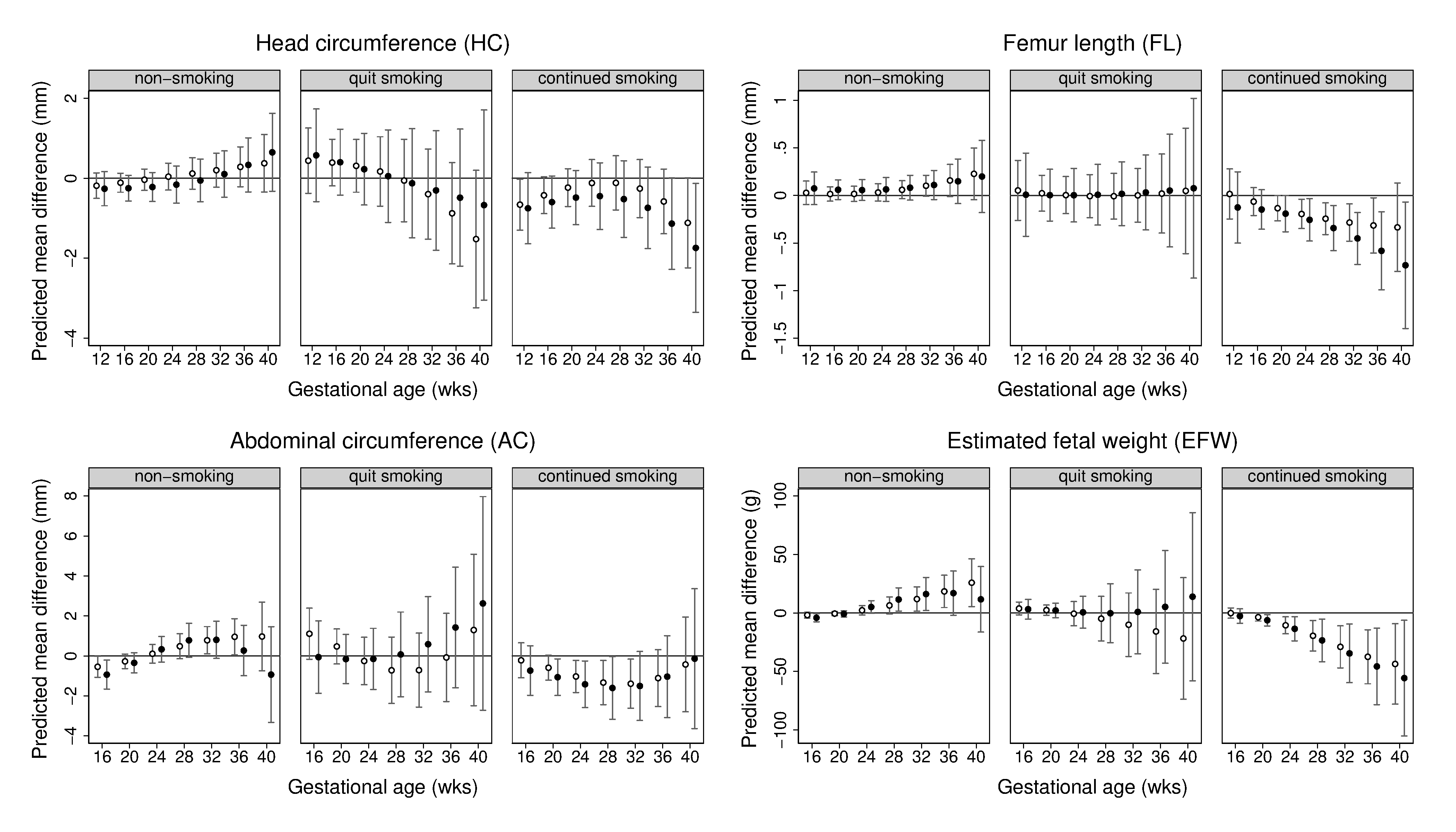

Supplement: S4 Fig — Predicted differences in mean HC (mm), FL (mm), AC (mm), and EFW (g) across gestation per maternal rs1051730 T allele increase in non-smokers, pre-pregnancy smokers who quit smoking before the second trimester, and pre-pregnancy smokers who continued smoking during pregnancy. Predicted mean differences (with 95% confidence intervals) in the pooled GenR and BiB cohort by analysis model: model 1 adjusting for cohort and maternal age (in white) and model 2 adjusting for cohort, maternal age, and fetal rs1051730 genotype (in black). (TIF) [file pmed.1002972.s004.tif]

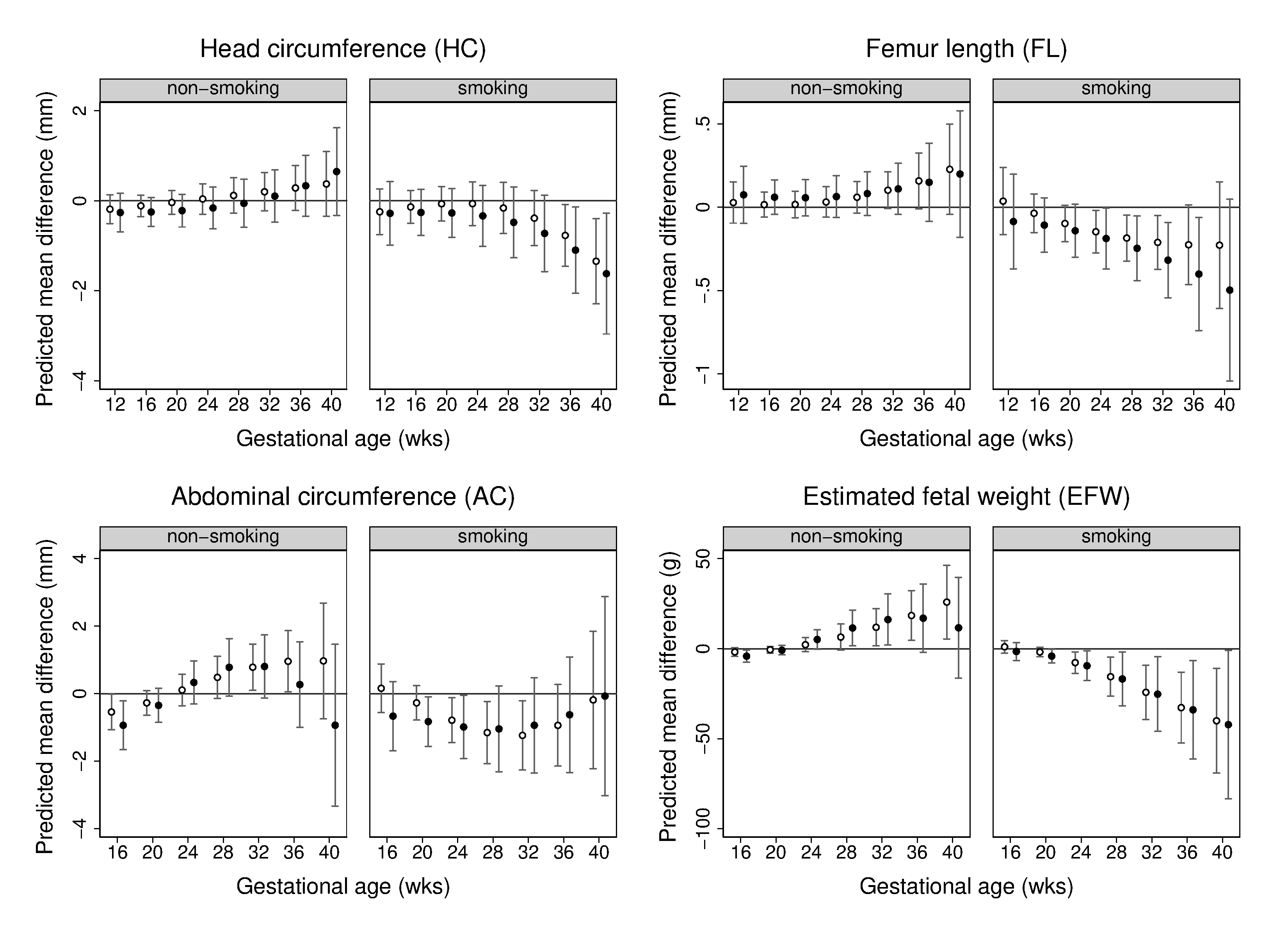

Supplement: S5 Fig — Predicted differences in mean HC (mm), FL (mm), AC (mm), and EFW (g) across gestation per maternal rs1051730 T allele increase in pre-pregnancy smokers and non-smokers. Predicted mean differences (with 95% confidence intervals) in the pooled GenR and BiB cohort by analysis model: model 1 adjusting for cohort and maternal age (in white) and model 2 adjusting for cohort, maternal age, and fetal rs1051730 genotype (in black). (TIF) [file pmed.1002972.s005.tif]

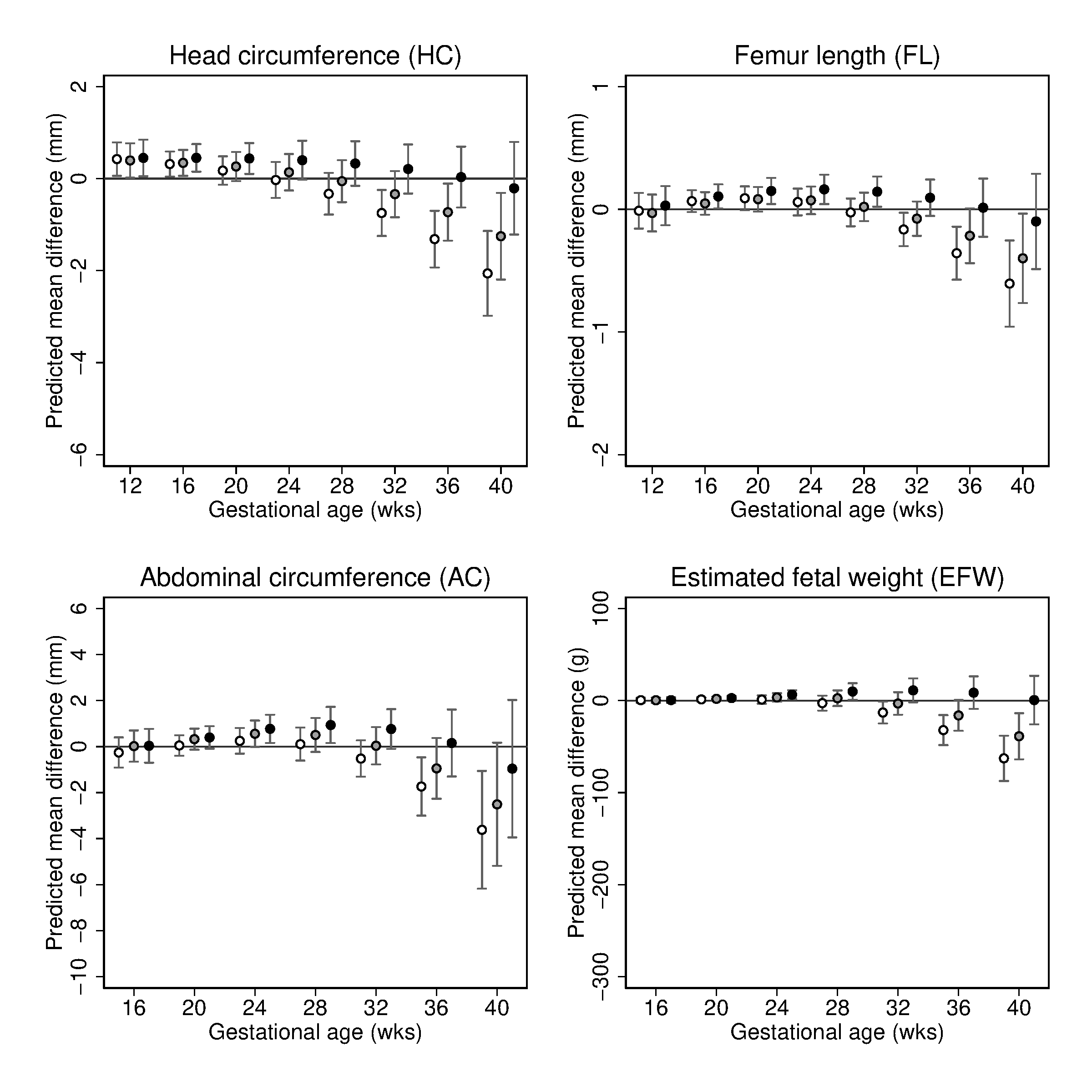

Supplement: S6 Fig — Predicted differences in mean HC (mm), FL (mm), AC (mm), and EFW (g) across gestation comparing partners who smoked during pregnancy to those who did not smoke during pregnancy (reference category). Predicted mean differences (with 95% confidence intervals) in the pooled GenR and BiB cohort by analysis model: model 1 adjusting for cohort only (in white); model 2 adjusting for cohort, infant sex, and partner age, height, body mass index, education level, and alcohol use during pregnancy (in grey); and model 3 adjusting for cohort; infant sex; partner age, height, body mass index, education level, and alcohol use during pregnancy; and smoking during pregnancy (in black). (TIF) [file pmed.1002972.s006.tif]

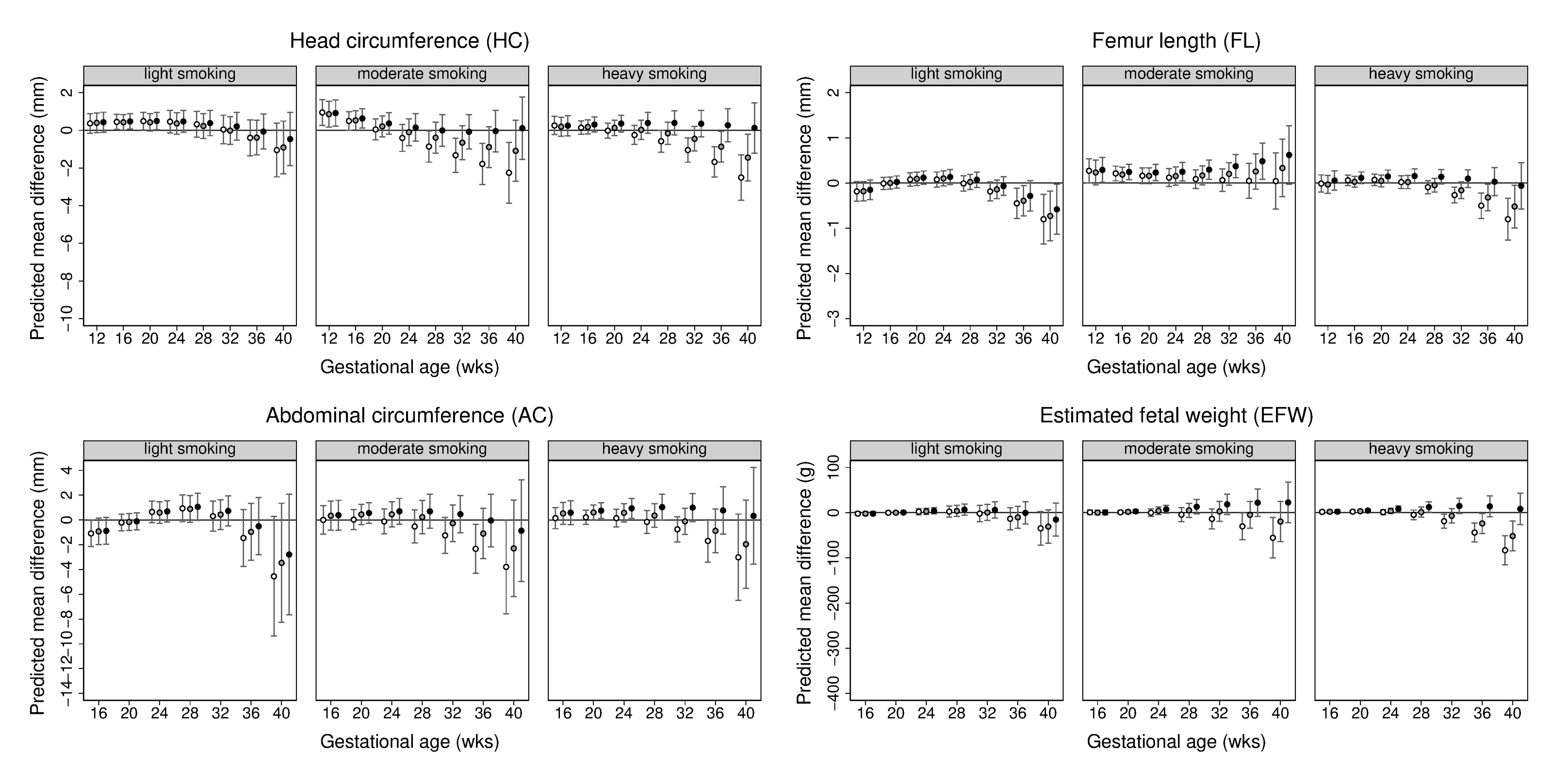

Supplement: S7 Fig — Predicted differences in mean HC (mm), FL (mm), AC (mm), and EFW (g) across gestation comparing partners who smoked during pregnancy by smoking intensity with those who did not smoke during pregnancy (reference category). Predicted mean differences (with 95% confidence intervals) in the pooled GenR and BiB cohort by analysis model: model 1 adjusting for cohort only (in white); model 2 adjusting for cohort, infant sex, and partner age, height, body mass index, education level, and alcohol use during pregnancy (in grey); and model 3 adjusting for cohort; infant sex; partner age, height, body mass index, education level, and alcohol use during pregnancy; and maternal smoking during pregnancy (in black). (TIF) [file pmed.1002972.s007.tif]

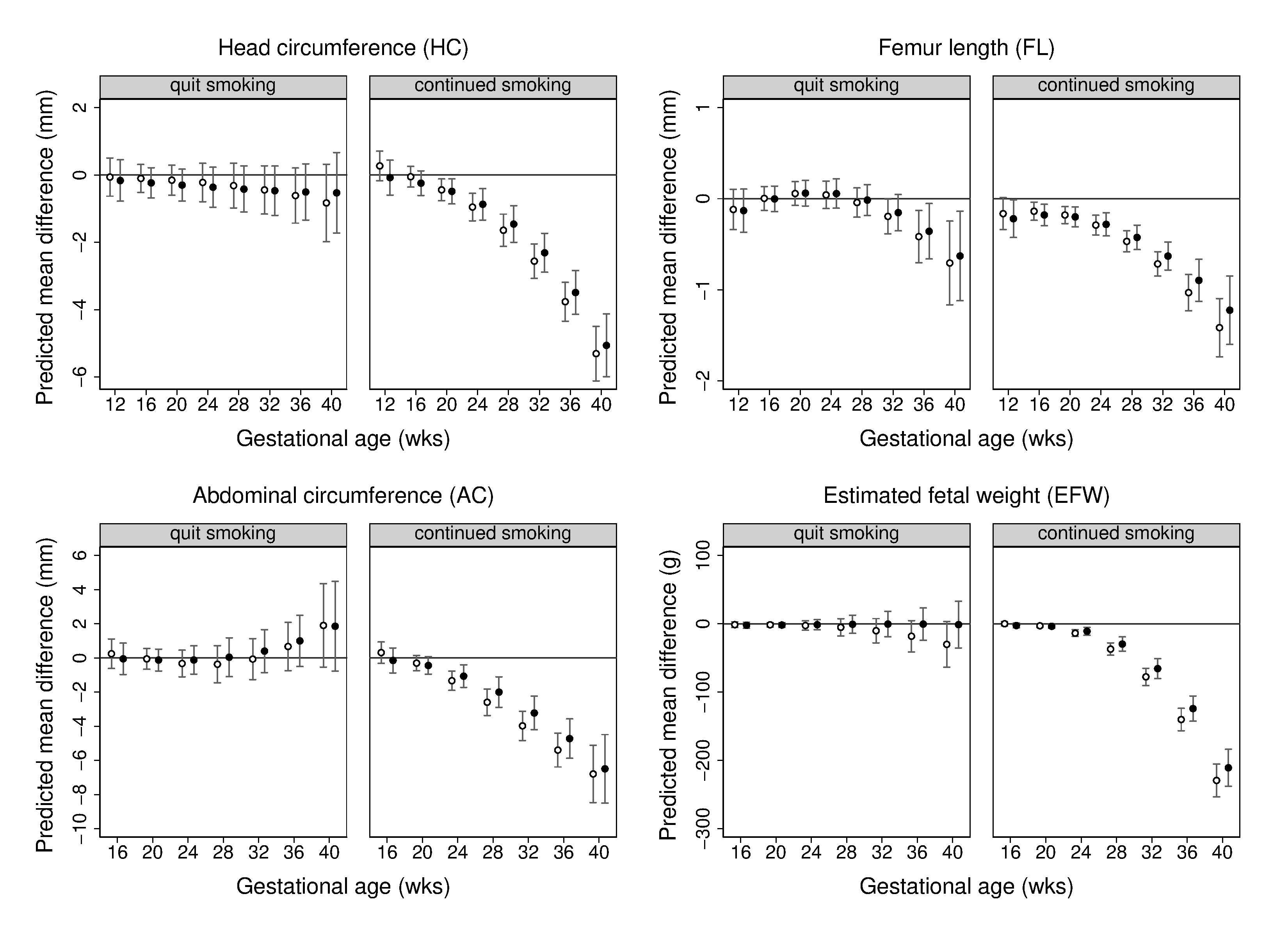

Supplement: S8 Fig — Predicted differences in mean HC (mm), FL (mm), AC (mm), and EFW (g) across gestation comparing pre-pregnancy smokers who quit smoking in early pregnancy and those who continued smoking during pregnancy with non-smokers (reference category)—complete case analysis. Predicted mean differences (with 95% confidence intervals) in the pooled GenR and BiB cohort by analysis model: model 1 adjusting for cohort only (in white) and model 2 adjusting for cohort, infant sex, and maternal age, parity, height, body mass index, education level, and alcohol use during pregnancy (in black). (TIF) [file pmed.1002972.s008.tif]

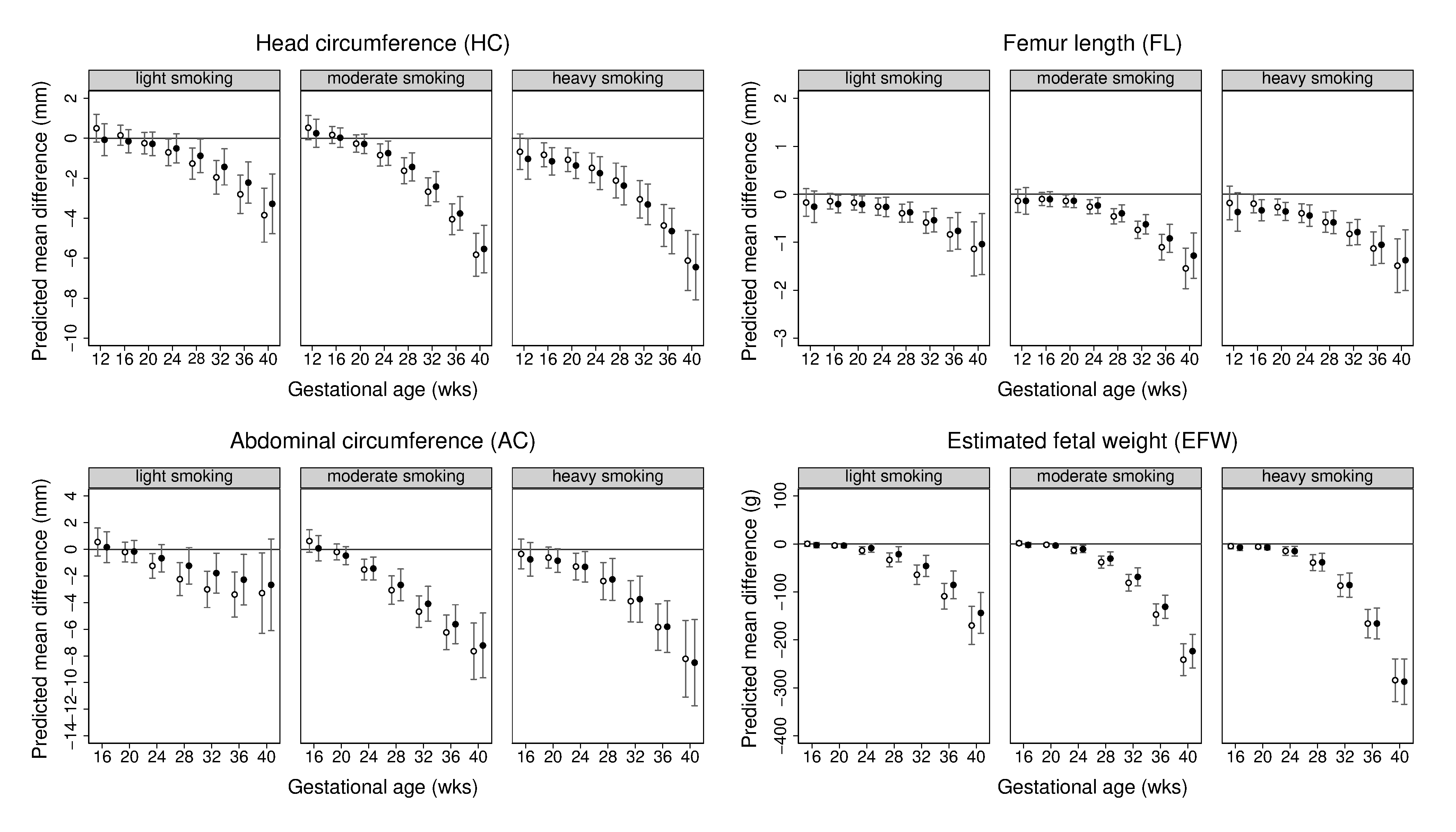

Supplement: S9 Fig — Predicted differences in mean HC (mm), FL (mm), AC (mm), and EFW (g) across gestation comparing pre-pregnancy smokers who continued smoking through pregnancy by smoking intensity with non-smokers (reference category)—complete case analysis. Predicted mean differences (with 95% confidence intervals) in the pooled GenR and BiB cohort by analysis model: model 1 adjusting for cohort only (in white) and model 2 adjusting for cohort, infant sex, and maternal age, parity, height, body mass index, education level, and alcohol use during pregnancy (in black). (TIF) [file pmed.1002972.s009.tif]

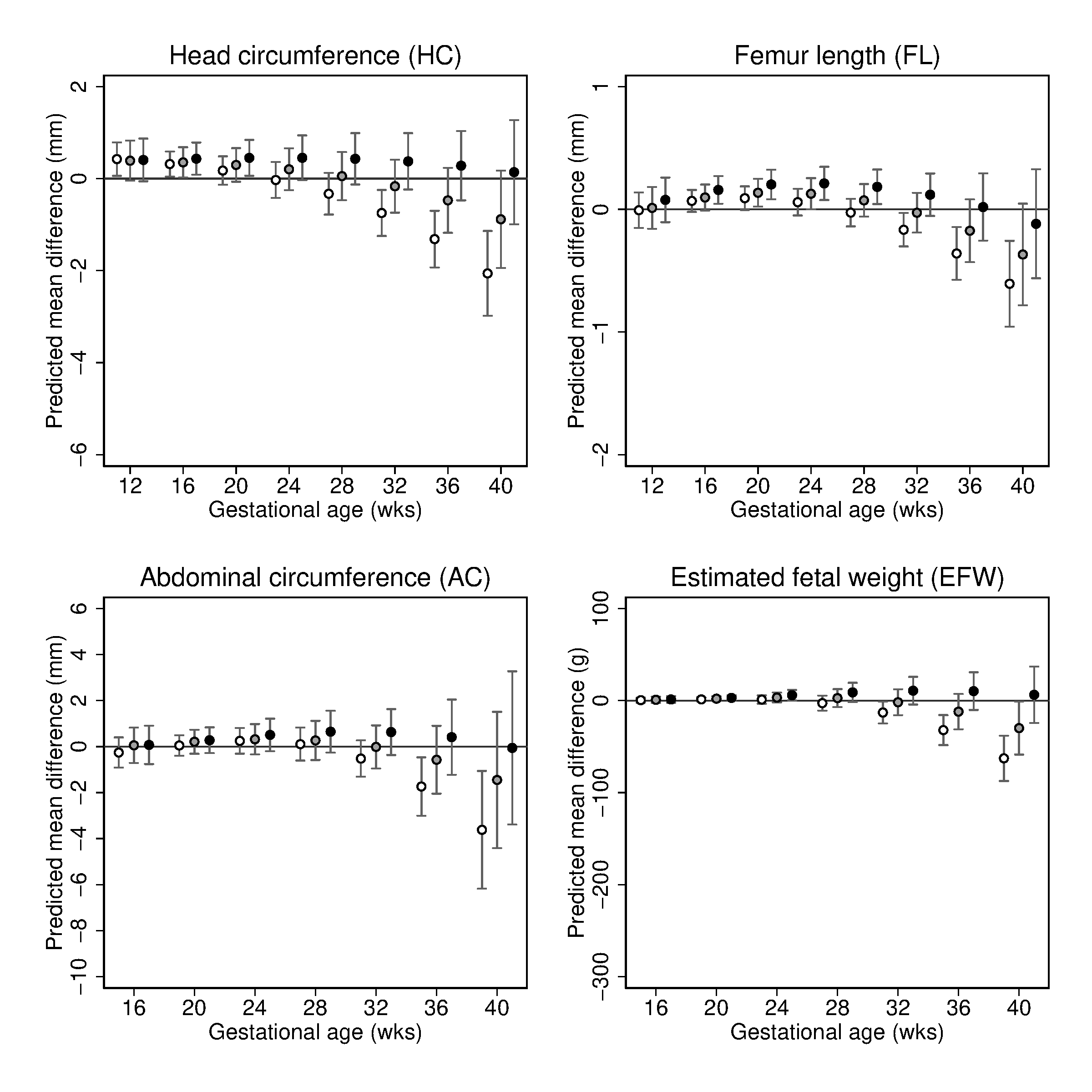

Supplement: S10 Fig — Predicted differences in mean HC (mm), FL (mm), AC (mm), and EFW (g) across gestation comparing partners who smoked during pregnancy with those who did not smoke during pregnancy (reference category)—complete case analysis. Predicted mean differences (with 95% confidence intervals) in the pooled GenR and BiB cohort by analysis model: model 1 adjusting for cohort only (in white); model 2 adjusting for cohort, infant sex, and partner age, height, body mass index, education level, and alcohol use during pregnancy (in grey); and model 3 adjusting for cohort; infant sex; partner age, height, body mass index, education level, and alcohol use during pregnancy; and maternal smoking during pregnancy (in black). (TIF) [file pmed.1002972.s010.tif]

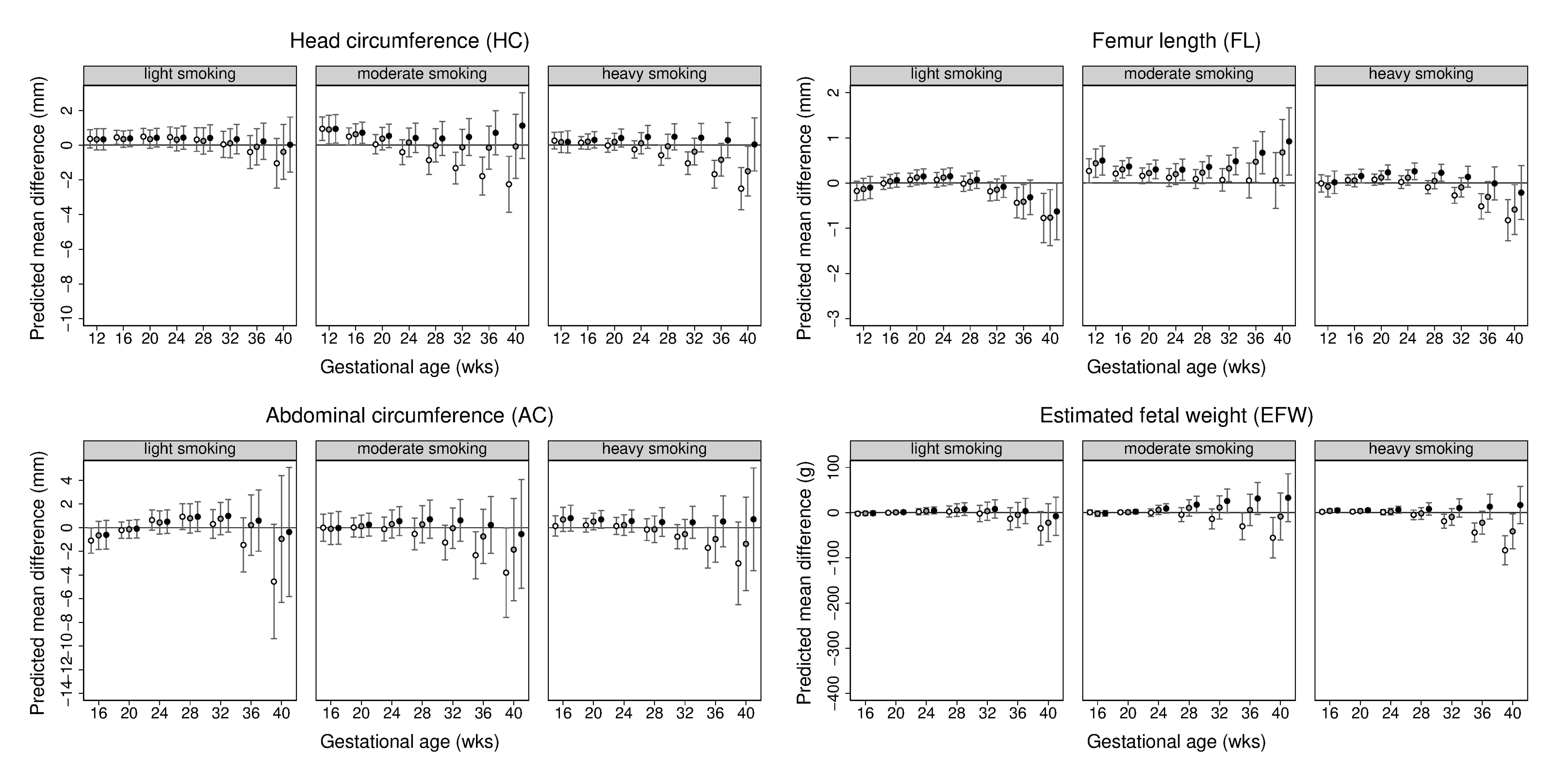

Supplement: S11 Fig — Predicted differences in mean HC (mm), FL (mm), AC (mm), and EFW (g) across gestation comparing partners who smoked during pregnancy by smoking intensity with those who did not smoke during pregnancy (reference category)—complete case analysis. Predicted mean differences (with 95% confidence intervals) in the pooled GenR and BiB cohort by analysis model: model 1 adjusting for cohort only (in white); model 2 adjusting for cohort, infant sex, and partner age, height, body mass index, education level, and alcohol use during pregnancy (in grey); and model 3 adjusting for cohort; infant sex; partner age, height, body mass index, education level, and alcohol use during pregnancy; and maternal smoking during pregnancy (in black). (TIF) [file pmed.1002972.s011.tif]

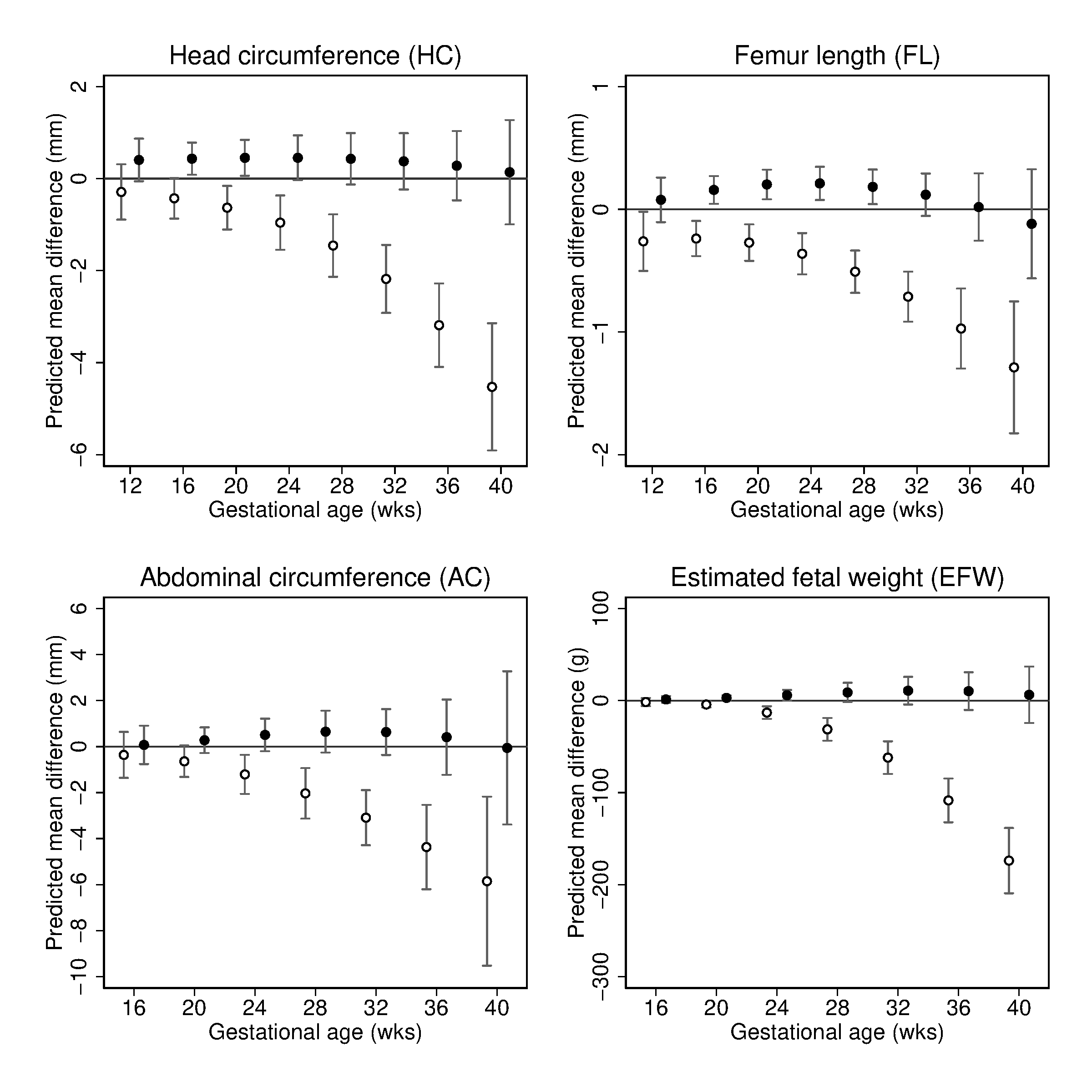

Supplement: S12 Fig — Predicted differences in mean HC (mm), FL (mm), AC (mm), and EFW (g) across gestation associated with maternal smoking (i.e., comparing maternal continued smoking through pregnancy with no maternal smoking during pregnancy [reference category]) and mother’s partner’s smoking (comparing partner smoking during pregnancy with no partner smoking during pregnancy [reference category])—complete case analysis. Predicted mean differences associated with maternal smoking (in white) and partner smoking (in black) are adjusted for cohort, infant sex, parity (for maternal smoking only), and respective parental age, height, BMI, education level, and alcohol use during pregnancy, and mutually adjusted for the smoking behaviour of the other parent. (TIF) [file pmed.1002972.s012.tif]
