# Supplementary material for: Associations of maternal quitting, reducing, and continuing smoking during pregnancy with longitudinal fetal growth: Findings from Mendelian randomization and parental negative control studies
Source: PLoS Med. 2019 Nov 13;16(11):e1002972. doi: 10.1371/journal.pmed.1002972 (PMC6853297; doi:10.1371/journal.pmed.1002972)
Supplement: S1 Table — (DOCX) [file pmed.1002972.s013.docx]

**S1 Table. Genotype quality control measures in GenR and BiB.**

| **Cohort** | **Genotyping method** | **If GWAS, directly genotyped or imputed** | **Call rate (%)** | **Risk allele frequency (T)** | **HWE**  **p value** | **% samples duplicated for genotyping** | **% concordance of duplicated samples** |
| --- | --- | --- | --- | --- | --- | --- | --- |
| GenR mothers | Taqman | - | 97.79 | 0.33 | 0.08 | 2.3 | 99.4 |
| GenR offspring | Illumina 660W Quad array | Directly genotyped | 99.96 | 0.32 | 0.18 | - | - |
| BiB mothers | Illumina HumanCoreExome chip | Directly genotyped | 99.95 | 0.32 | 0.87 | - | - |
| BiB offspring | Illumina HumanCoreExome chip | Directly genotyped | 99.95 | 0.33 | 0.50 | - | - |

Abbreviations: GWAS = genome wide association study; HEW = Hardy Weinberg equilibrium.
