# Supplementary material for: Associations of maternal quitting, reducing, and continuing smoking during pregnancy with longitudinal fetal growth: Findings from Mendelian randomization and parental negative control studies
Source: PLoS Med. 2019 Nov 13;16(11):e1002972. doi: 10.1371/journal.pmed.1002972 (PMC6853297; doi:10.1371/journal.pmed.1002972)
Supplement: S2 Table — (DOCX) [file pmed.1002972.s014.docx]

**S2 Table. Descriptive statistics of the repeat ultrasound and birth anthropometric measurements in GenR and BiB.**

| **Measure** | **N with at least one measure** | **Median (range) no. of**  **measurements per individual** | **Total no. of**  **measurements** | **Mean (SD)** | **Median (range) gestational age at measurement (wks)** | **No (%) of birth measurements** |
| --- | --- | --- | --- | --- | --- | --- |
| **GenR** |  |  |  |  |  |  |
| Head circumference (mm) | 4292 | 3 (1; 4) | 13216 | 224.8 (88.4) | 23.3 (12.0-43.4) | 2390 (18.1) |
| Femur length (mm) | 4277 | 3 (1; 3) | 10529 | 38.5 (17.5) | 21.1 (12.0-38.1) | 0 (0.0) |
| Abdominal circumference (mm) | 4262 | 2 (1; 2) | 8275 | 211.3 (56.1) | 27.8 (18.0-38.1) | 0 (0.0) |
| Estimated fetal weight (g) | 4680 | 3 (1; 3) | 12902 | 1899.5 (1337.4) | 30.5 (18.0-43.4) | 4660 (36.1) |
| **BiB** |  |  |  |  |  |  |
| Head circumference (mm) | 3936 | 3 (1; 12) | 11867 | 261.6 (83.4) | 32.3 (12.0-42.9) | 3612 (30.4) |
| Femur length (mm) | 3933 | 3 (1; 11) | 8416 | 44.1 (17.2) | 21.0 (12.0-42.7) | 0 (0.0) |
| Abdominal circumference (mm) | 3874 | 3 (1; 10) | 10314 | 255.2 (76.9) | 34.0 (16.0-42.9) | 3402 (33.0) |
| Estimated fetal weight (g) | 3938 | 3 (1; 9) | 10497 | 2065.7 (1346.2) | 34.1 (16.0-42.9) | 3938 (37.5) |

Abbreviations: SD = standard deviation; wks = weeks.
