# Supplementary material for: Associations of maternal quitting, reducing, and continuing smoking during pregnancy with longitudinal fetal growth: Findings from Mendelian randomization and parental negative control studies
Source: PLoS Med. 2019 Nov 13;16(11):e1002972. doi: 10.1371/journal.pmed.1002972 (PMC6853297; doi:10.1371/journal.pmed.1002972)
Supplement: S3 Table — (DOCX) [file pmed.1002972.s015.docx]

**S3 Table. Comparison of observed means of fetal size with those predicted by the multilevel fractional polynomial model in GenR and BiB.**

| **GenR** |  |  | **Observed** | | **Predicted** | | **Difference** | | **95% limits of agreement** | |
| --- | --- | --- | --- | --- | --- | --- | --- | --- | --- | --- |
| **Measure** | **Gestational age** | **No. of obs** | **Mean** | **SD** | **Mean** | **SD** | **Mean** | **SD** | **P5** | **P95** |
| Head circumference (mm) | 12-15 wks | 1962 | 84.3 | 11.7 | 84.1 | 11.3 | 0.2 | 2.4 | -3.5 | 4.2 |
|  | 16-19 wks | 539 | 141.9 | 14.9 | 141.6 | 13.7 | 0.3 | 3.2 | -4.7 | 5.5 |
|  | 20-23 wks | 2804 | 183.1 | 10.3 | 183.9 | 9.1 | -0.8 | 3.5 | -6.4 | 4.8 |
|  | 24-27 wks | 42 | 227.1 | 11.2 | 228.5 | 9.3 | -1.5 | 4.6 | -10.7 | 5.1 |
|  | 28-31 wks | 3117 | 282.8 | 10.2 | 281.7 | 7.1 | 1.1 | 5.4 | -8.0 | 9.7 |
|  | 32-35 wks | 276 | 302.7 | 11.5 | 302.5 | 7.5 | 0.2 | 6.3 | -9.8 | 8.7 |
|  | 36-39 wks | 454 | 332.6 | 16.7 | 334.0 | 9.1 | -1.4 | 8.7 | -16.3 | 12.6 |
|  | >39 wks | 1927 | 341.7 | 15.7 | 342.4 | 9.2 | 1.1 | 7.2 | -12.6 | 10.5 |
| Femur length (mm) | 12-15 wks | 1623 | 10.2 | 2.8 | 10.3 | 2.9 | 0.0 | 1.2 | -1.9 | 2.1 |
|  | 16-19 wks | 549 | 24.3 | 4.1 | 24.3 | 3.4 | 0.0 | 1.3 | -2.0 | 2.1 |
|  | 20-23 wks | 2800 | 34.2 | 2.6 | 34.2 | 2.3 | 0.0 | 1.0 | -1.6 | 1.8 |
|  | 24-27 wks | 42 | 44.6 | 3.1 | 44.6 | 2.6 | 0.0 | 1.0 | -1.9 | 1.9 |
|  | 28-31 wks | 3145 | 56.7 | 2.5 | 56.7 | 1.9 | 0.0 | 0.8 | -1.4 | 1.4 |
|  | 32-35 wks | 276 | 61.7 | 2.7 | 61.7 | 2.1 | 0.0 | 0.8 | -1.3 | 1.4 |
|  | 36-39 wks | 5 | 69.3 | 4.8 | 69.5 | 3.9 | -0.2 | 0.9 | -1.8 | 0.5 |
|  | >39 wks | 0 | - | - | - | - | - | - | - | - |
| Abdominal circumference (mm) | 16-19 wks | 217 | 136.1 | 7.8 | 137.3 | 3.6 | -1.2 | 5.5 | -10.3 | 8.1 |
|  | 20-23 wks | 2804 | 160.7 | 11.3 | 160.6 | 8.8 | 0.2 | 5.4 | -8.5 | 9.3 |
|  | 24-27 wks | 42 | 203.8 | 15.5 | 203.6 | 11.5 | 0.2 | 5.9 | -10.4 | 10.3 |
|  | 28-31 wks | 3136 | 261.1 | 13.9 | 261.3 | 8.4 | -0.2 | 9.5 | -16.2 | 15.0 |
|  | 32-35 wks | 276 | 285.6 | 16.1 | 284.4 | 6.8 | 1.2 | 14.9 | -20.9 | 25.0 |
|  | 36-39 wks | 5 | 324.8 | 26.6 | 313.7 | 4.5 | 11.1 | 23.9 | -26.3 | 28.5 |
|  | >39 wks | 0 | - | - | - | - | - | - | - | - |
| Estimated fetal weight (g) | 16-19 wks | 213 | 263.3 | 33.4 | 253.8 | 18.0 | 9.4 | 25.5 | -30.1 | 58.3 |
|  | 20-23 wks | 2786 | 399.5 | 68.6 | 403.4 | 63.1 | -3.9 | 24.3 | -42.3 | 38.0 |
|  | 24-27 wks | 44 | 765.5 | 146.4 | 779.9 | 119.1 | -14.4 | 56.8 | -120.9 | 59.8 |
|  | 28-31 wks | 3143 | 1557.3 | 200.0 | 1551 | 158.6 | 6.1 | 84.9 | -128.1 | 147.9 |
|  | 32-35 wks | 337 | 2066.1 | 326.9 | 2043.1 | 248.1 | 23.0 | 137.3 | -207.0 | 250.3 |
|  | 36-39 wks | 973 | 3137.7 | 490.5 | 3124.0 | 353.7 | 13.7 | 202.8 | -311.0 | 350.5 |
|  | >39 wks | 3564 | 3613.1 | 466.9 | 3628.5 | 335.3 | 6.1 | 230.2 | -383.2 | 367.7 |

**S3 Table. *Continued.***

| **BiB** |  |  | **Observed** | | **Predicted** | | **Difference** | | **95% limits of agreement** | |
| --- | --- | --- | --- | --- | --- | --- | --- | --- | --- | --- |
| **Measure** | **Gestational age** | **No. of obs** | **Mean** | **SD** | **Mean** | **SD** | **Mean** | **SD** | **P5** | **P95** |
| Head circumference (mm) | 12-15 wks | 486 | 97.2 | 15.1 | 97.3 | 14.6 | -0.1 | 2.0 | -3.6 | 3.0 |
|  | 16-19 wks | 1567 | 166.5 | 14.3 | 165.9 | 13.6 | 0.6 | 2.9 | -3.7 | 5.5 |
|  | 20-23 wks | 2899 | 181.9 | 11.5 | 182.4 | 10.8 | -0.5 | 2.8 | -5.0 | 4.0 |
|  | 24-27 wks | 181 | 242.4 | 20.7 | 242.0 | 18.8 | 0.3 | 4.9 | -7.6 | 8.7 |
|  | 28-31 wks | 737 | 286.6 | 13.7 | 285.1 | 12.1 | 1.5 | 5.2 | -6.9 | 9.7 |
|  | 32-35 wks | 1845 | 316.4 | 11.8 | 316.4 | 10.0 | 0.0 | 5.2 | -8.6 | 8.4 |
|  | 36-39 wks | 2189 | 339.7 | 14.2 | 340.1 | 10.6 | -0.4 | 5.7 | -9.5 | 9.2 |
|  | >39 wks | 1963 | 349.8 | 13.2 | 349.6 | 8.9 | 1.5 | 5.4 | -8.2 | 8.7 |
| Femur length (mm) | 12-15 wks | 404 | 13.7 | 3.4 | 13.8 | 3.3 | -0.1 | 1.1 | -2.0 | 1.8 |
|  | 16-19 wks | 1588 | 29.9 | 3.5 | 29.7 | 3.1 | 0.2 | 1.1 | -1.4 | 2.2 |
|  | 20-23 wks | 2949 | 33.5 | 2.7 | 33.6 | 2.4 | -0.1 | 1.0 | -1.8 | 1.7 |
|  | 24-27 wks | 181 | 47.0 | 4.7 | 47.1 | 4.0 | -0.1 | 1.4 | -2.2 | 2.2 |
|  | 28-31 wks | 740 | 56.8 | 3.3 | 56.8 | 2.7 | 0.0 | 1.4 | -2.2 | 2.2 |
|  | 32-35 wks | 1823 | 64.8 | 2.7 | 64.7 | 2.2 | 0.1 | 1.1 | -1.7 | 2.0 |
|  | 36-39 wks | 714 | 69.7 | 3.0 | 69.8 | 2.4 | -0.1 | 1.1 | -1.8 | 1.9 |
|  | >39 wks | 17 | 72.3 | 2.7 | 72.6 | 2.4 | 0.0 | 1.0 | -2.1 | 1.3 |
| Abdominal circumference (mm) | 16-19 wks | 1095 | 147.3 | 8.3 | 146.7 | 5.4 | 0.6 | 4.3 | -6.1 | 7.3 |
|  | 20-23 wks | 2342 | 156.3 | 11.2 | 156.6 | 9.3 | -0.3 | 4.1 | -6.9 | 6.7 |
|  | 24-27 wks | 164 | 216.7 | 21.2 | 216.7 | 19.3 | 0.0 | 5.5 | -9.6 | 10.0 |
|  | 28-31 wks | 758 | 260.7 | 19.4 | 261.7 | 16.7 | -1.0 | 6.3 | -10.8 | 8.9 |
|  | 32-35 wks | 1906 | 298.3 | 18.9 | 297.0 | 14.2 | 1.2 | 8.2 | -11.3 | 14.2 |
|  | 36-39 wks | 2185 | 319.0 | 23.6 | 319.6 | 12.7 | -0.6 | 15.3 | -27.5 | 22.8 |
|  | >39 wks | 1864 | 324.8 | 22.9 | 325.3 | 8.7 | -1.0 | 16.0 | -28.0 | 24.5 |
| Estimated fetal weight (g) | 16-19 wks | 1059 | 316.2 | 34.2 | 312.5 | 29.2 | 3.7 | 9.6 | -9.8 | 21.4 |
|  | 20-23 wks | 2246 | 369.6 | 68.1 | 373.3 | 68.1 | -3.7 | 13.9 | -27.0 | 16.9 |
|  | 24-27 wks | 166 | 917.5 | 234.1 | 931.5 | 223.6 | -14.0 | 48.1 | -83.6 | 73.4 |
|  | 28-31 wks | 735 | 1564.7 | 284.8 | 1567.8 | 255.1 | -3.1 | 80.0 | -127.6 | 130.7 |
|  | 32-35 wks | 1844 | 2339.4 | 339.6 | 2298.1 | 290.0 | 41.4 | 114.9 | -130.6 | 229.5 |
|  | 36-39 wks | 2316 | 3165.0 | 479.5 | 3158.6 | 397.2 | 6.4 | 166.7 | -258.7 | 285.9 |
|  | >39 wks | 2131 | 3553.2 | 453.7 | 3599.7 | 338.6 | -3.1 | 164.4 | -312.7 | 220.0 |

Comparison of predicted values of fetal size by the multilevel fractional polynomial models with the actual values observed; The 95% limits of agreement refer to the range within which 95% of the differences between the predicted and observed values lie. Abbreviations: SD = standard deviation; No. of obs = number of observed measurements; p5 = 5^th^ percentile and p95 = 95^th^ percentile.
