# Supplementary material for: Associations of maternal quitting, reducing, and continuing smoking during pregnancy with longitudinal fetal growth: Findings from Mendelian randomization and parental negative control studies
Source: PLoS Med. 2019 Nov 13;16(11):e1002972. doi: 10.1371/journal.pmed.1002972 (PMC6853297; doi:10.1371/journal.pmed.1002972)
Supplement: S4 Table — (DOCX) [file pmed.1002972.s016.docx]

**S4 Table. Participant characteristics by availability of maternal rs1051730 genotype and partner smoking data.**

| **GenR** | **All**  **(N = 4682)** | **With maternal genotype data (N = 3604)** | **With partner smoking data**  **(N = 4206)** |
| --- | --- | --- | --- |
| **Infant characteristics** |  |  |  |
| Sex, % (N) |  |  |  |
| Male | 50.0 (2339) | 49.7 (1792) | 49.7 (2091) |
| Female | 50.0 (2343) | 50.3 (1812) | 50.3 (2115) |
| Birth weight (g), mean (SD) | 3462.4 (568.4) | 3470.6 (555.4) | 3464.1 (548.6) |
| Missing, % (N) | 0.5 (22) | 0.5 (17) | 0.5 (22) |
| Gestational age at birth (weeks), mean (SD) | 39.9 (1.8) | 39.9 (1.8) | 39.9 (1.8) |
| Missing, % (N) | 0.0 (2) | 0.1 (2) | 0.0 (2) |
| **Maternal characteristics** |  |  |  |
| Age (yrs), mean (SD) | 31.3 (4.6) | 31.1 (4.5) | 31.1 (4.5) |
| Height (cm), mean (SD) | 170.3 (6.5) | 170.2 (6.5) | 170.2 (6.5) |
| Missing, % (N) | 8.4 (394) | 0.0 (1) | 0.0 (1) |
| Body mass index (kg/m^2^), mean (SD) | 23.2 (4.0) | 23.2 (4.0) | 23.2 (4.0) |
| Missing, % (N) | 18.3 (856) | 11.0 (398) | 10.5 (442) |
| Multiparity, % (N) |  |  |  |
| Primiparous | 59.0 (2694) | 59.7 (2148) | 59.8 (2512) |
| 1 | 31.4 (1432) | 30.9 (1113) | 30.9 (1297) |
| 2 | 7.9 (361) | 7.9 (284) | 7.7 (323) |
| ≥ 3 | 1.7 (76) | 1.4 (52) | 1.6 (68) |
| Missing | 2.5 (119) | 0.2 (7) | 0.1 (6) |
| Education, % (N) |  |  |  |
| Low | 4.4 (205) | 4.4 (159) | 4.5 (190) |
| Intermediate | 38.4 (1779) | 38.7 (1382) | 38.5 (1611) |
| High | 57.2 (2647) | 56.9 (2033) | 56.9 (2380) |
| Missing | 1.1 (51) | 0.8 (30) | 0.6 (25) |
| Smoking during pregnancy, % (N) |  |  |  |
| Non-smoker | 73.9 (3461) | 72.8 (2622) | 73.6 (3097) |
| Pre-pregnancy smoker quitting smoking  before the second trimester | 9.0 (423) | 9.2 (332) | 9.4 (394) |
| Pre-pregnancy smoker continuing  smoking during pregnancy | 17.0 (798) | 18.0 (650) | 17.0 (715) |
| Alcohol during pregnancy, % (N) |  |  |  |
| No | 35.4 (1501) | 34.6 (1233) | 35.3 (1483) |
| Yes | 64.6 (2740) | 65.4 (2328) | 64.7 (2714) |
| Missing | 9.4 (441) | 1.2 (43) | 0.2 (9) |

**S4 Table. *Continued.***

| **BiB** | **All**  **(N = 3939)** | **With maternal genotype data (N = 2923)** | **With partner smoking data**  **(N = 1331)** |
| --- | --- | --- | --- |
| **Infant characteristics** |  |  |  |
| Sex, % (N) |  |  |  |
| Male | 51.9 (2046) | 51.6 (1509) | 51.9 (691) |
| Female | 48.1 (1893) | 48.4 (1414) | 48.1 (640) |
| Birth weight (g), mean (SD) | 3371.0 (543.1) | 3372.8 (542.3) | 3402.0 (517.0) |
| Missing, % (N) | 0.0 (1) | 0.0 (1) | 0 (0) |
| Gestational age at birth (weeks), mean (SD) | 39.3 (1.8) | 39.3 (1.8) | 39.4 (1.6) |
| Missing, % (N) | 0.0 (0) | 0.0 (0) | 0.0 (0) |
| **Maternal characteristics** |  |  |  |
| Age (yrs), mean (SD) | 26.7 (6.0) | 26.8 (6.0) | 26.9 (5.8) |
| Height (cm), mean (SD) | 164.1 (6.2) | 164.3 (6.2) | 164.6 (6.1) |
| Missing, % (N) | 1.6 (63) | 1.4 (41) | 1.5 (20) |
| Body mass index (kg/m^2^), mean (SD) | 26.6 (6.0) | 26.7 (6.0) | 26.6 (5.9) |
| Missing, % (N) | 4.8 (190) | 4.1 (120) | 4.4 (59) |
| Multiparity, % (N) |  |  |  |
| Primiparous | 49.7 (1898) | 49.6 (1414) | 54.7 (714) |
| 1 | 30.5 (1164) | 30.7 (875) | 30.3 (396) |
| 2 | 12.9 (491) | 12.7 (361) | 10.5 (137) |
| ≥ 3 | 7.0 (266) | 7.1 (202) | 4.4 (58) |
| Missing | 3.0 (120) | 2.4 (71) | 2.0 (26) |
| Education, % (N) |  |  |  |
| Low | 21.8 (772) | 21.8 (578) | 16.6 (200) |
| Intermediate | 36.6 (1300) | 36.7 (972) | 36.9 (444) |
| High | 41.6 (1476) | 41.4 (1096) | 46.4 (558) |
| Missing | 9.9 (391) | 9.5 (277) | 9.7 (129) |
| Smoking during pregnancy, % (N) |  |  |  |
| Non-smoker | 59.1 (2328) | 57.3 (1675) | 62.7 (834) |
| Pre-pregnancy smoker quitting smoking  before the second trimester | 10.8 (427) | 12.2 (356) | 12.6 (168) |
| Pre-pregnancy smoker continuing  smoking during pregnancy | 30.1 (1184) | 30.5 (892) | 24.7 (329) |
| Alcohol during pregnancy, % (N) |  |  |  |
| No | 34.6 (1362) | 35.5 (1036) | 29.9 (397) |
| Yes | 65.4 (2569) | 64.5 (1882) | 70.1 (931) |
| Missing | 0.2 (8) | 0.2 (5) | 0.2 (3) |

Distribution of infant and maternal characteristics in the total GenR and BiB cohort and sub-cohorts with partner smoking and maternal genotype data. For all variables, mean (SD) or percentages (N) are only given for singletons with no missing values.
