# Supplementary material for: Associations of maternal quitting, reducing, and continuing smoking during pregnancy with longitudinal fetal growth: Findings from Mendelian randomization and parental negative control studies
Source: PLoS Med. 2019 Nov 13;16(11):e1002972. doi: 10.1371/journal.pmed.1002972 (PMC6853297; doi:10.1371/journal.pmed.1002972)
Supplement: S5 Table — (DOCX) [file pmed.1002972.s017.docx]

**S5 Table. Participant characteristics by maternal smoking status during pregnancy.**

| **GenR** | **Non-smokers**  **(N = 3461)** | **Pre-pregnancy smokers quitting smoking in early pregnancy**  **(N = 423)** | **Pre-pregnancy smokers continuing smoking during pregnancy**  **(N = 798)** |
| --- | --- | --- | --- |
| **Infant characteristics** |  |  |  |
| Sex, % (N) |  |  |  |
| Male | 49.3 (1705) | 48.7 (206) | 53.6 (428) |
| Female | 50.7 (1756) | 51.3 (217) | 46.4 (370) |
| Birth weight (g), mean (SD) | 3499.7 (555.9) | 3564.2 (588.4) | 3256.4 (571.5) |
| Missing, % (N) | 0.4 (13) | 0.5 (2) | 0.9 (7) |
| Gestational age at birth (weeks), mean (SD) | 39.9 (1.8) | 39.9 (1.7) | 39.7 (1.8) |
| Missing, % (N) | 0.0 (1) | 0.2 (1) | 0.0 (0) |
| **Maternal characteristics** |  |  |  |
| Age (yrs), mean (SD) | 31.7 (4.2) | 30.8 (4.5) | 29.8 (5.6) |
| Height (cm), mean (SD) | 170.5 (6.5) | 170.4 (6.5) | 169.2 (6.6) |
| Missing, % (N) | 9.7 (336) | 6.1 (26) | 4.0 (32) |
| Body mass index (kg/m^2^), mean (SD) | 23.2 (3.9) | 22.8 (3.6) | 23.2 (4.4) |
| Missing, % (N) | 19.2 (663) | 14.9 (63) | 16.3 (130) |
| Multiparity, % (N) |  |  |  |
| Primiparous | 58.2 (1956) | 71.0 (292) | 56.5 (446) |
| 1 | 32.6 (1096) | 22.6 (93) | 30.8 (243) |
| 2 | 7.7 (258) | 5.1 (21) | 10.4 (82) |
| ≥ 3 | 1.6 (53) | 1.2 (5) | 2.3 (18) |
| Missing | 2.8 (98) | 2.8 (12) | 1.1 (9) |
| Education, % (N) |  |  |  |
| Low | 2.3 (78) | 5.0 (21) | 13.7 (106) |
| Intermediate | 33.1 (1137) | 40.9 (172) | 60.7 (470) |
| High | 64.6 (2221) | 54.2 (228) | 25.6 (198) |
| Missing | 0.7 (25) | 0.5 (2) | 3.0 (24) |
| Alcohol during pregnancy, % (N) |  |  |  |
| No | 36.8 (1151) | 17.4 (69) | 39.0 (281) |
| Yes | 63.2 (1973) | 82.6 (328) | 61.0 (439) |
| Missing | 9.7 (337) | 6.1 (26) | 9.8 (78) |

**S5 Table. *Continued.***

| **BiB** | **Non-smokers**  **(N = 2328)** | **Pre-pregnancy smokers quitting smoking in early pregnancy**  **(N = 427)** | **Pre-pregnancy smokers continuing smoking during pregnancy**  **(N = 1184)** |
| --- | --- | --- | --- |
| **Infant characteristics** |  |  |  |
| Sex, % (N) |  |  |  |
| Male | 51.0 (1188) | 55.3 (236) | 52.5 (622) |
| Female | 49.0 (1140) | 44.7 (191) | 47.5 (562) |
| Birth weight (g), mean (SD) | 3461.0 (515.9) | 3444.7 (555.9) | 3167.6 (536.4) |
| Missing, % (N) | 0.0 (0) | 0.2 (1) | 0.0 (0) |
| Gestational age at birth (weeks), mean (SD) | 39.4 (1.7) | 39.5 (1.8) | 39.1 (1.9) |
| Missing, % (N) | 0.0 (0) | 0.0 (0) | 0.0 (0) |
| **Maternal characteristics** |  |  |  |
| Age (yrs), mean (SD) | 28.2 (5.8) | 25.5 (5.6) | 24.4 (5.5) |
| Height (cm), mean (SD) | 164.3 (6.1) | 164.5 (6.3) | 163.6 (6.3) |
| Missing, % (N) | 1.5 (36) | 1.4 (6) | 1.8 (21) |
| Body mass index (kg/m^2^), mean (SD) | 26.8 (6.0) | 26.6 (5.7) | 26.0 (6.0) |
| Missing, % (N) | 4.9 (115) | 2.6 (11) | 5.4 (64) |
| Multiparity, % (N) |  |  |  |
| Primiparous | 49.1 (1106) | 63.6 (267) | 45.9 (525) |
| 1 | 33.1 (745) | 21.4 (90) | 28.7 (329) |
| 2 | 12.2 (276) | 9.5 (40) | 15.3 (175) |
| ≥ 3 | 5.6 (127) | 5.5 (23) | 10.1 (116) |
| Missing | 3.2 (74) | 1.6 (7) | 3.3 (39) |
| Education, % (N) |  |  |  |
| Low | 12.9 (266) | 20.2 (79) | 39.3 (427) |
| Intermediate | 31.7 (656) | 43.7 (171) | 43.5 (473) |
| High | 55.5 (1148) | 36.1 (141) | 17.2 (187) |
| Missing | 11.1 (258) | 8.4 (36) | 8.2 (97) |
| Alcohol during pregnancy, % (N) |  |  |  |
| No | 36.1 (837) | 27.4 (117) | 34.5 (408) |
| Yes | 63.9 (1484) | 72.6 (310) | 65.5 (775) |
| Missing | 0.3 (7) | 0.0 (0) | 0.1 (1) |

Distribution of infant and maternal characteristics by maternal smoking status during pregnancy in GenR and BiB. For all variables, mean (SD) or percentages (N) are only given for singletons with no missing values.
