# Supplementary material for: Associations of maternal quitting, reducing, and continuing smoking during pregnancy with longitudinal fetal growth: Findings from Mendelian randomization and parental negative control studies
Source: PLoS Med. 2019 Nov 13;16(11):e1002972. doi: 10.1371/journal.pmed.1002972 (PMC6853297; doi:10.1371/journal.pmed.1002972)
Supplement: S7 Table — (DOCX) [file pmed.1002972.s019.docx]

**S7 Table. Maternal smoking during pregnancy and predicted differences in mean fetal size (with 95% CIs) across gestation, overall and stratified by cohort.**

|  | **Predicted mean difference (95% CI)** | | | | | | | |
| --- | --- | --- | --- | --- | --- | --- | --- | --- |
| **HC (mm)** | **12 wks** | **16 wks** | **20 wks** | **24 wks** | **28 wks** | **32 wks** | **36 wks** | **40 wks** |
| Maternal smoking during pregnancy |  |  |  |  |  |  |  |  |
| All |  |  |  |  |  |  |  |  |
| Non-smoking | REF | REF | REF | REF | REF | REF | REF | REF |
| Quit smoking in early pregnancy | -0.11 (-0.69; 0.46) | -0.17 (-0.59; 0.25) | -0.22 (-0.67; 0.22) | -0.27 (-0.83; 0.29) | -0.31 (-0.96; 0.33) | -0.34 (-1.03; 0.35) | -0.36 (-1.14; 0.43) | -0.36 (-1.48; 0.76) |
| Continued smoking during pregnancy | 0.12 (-0.35; 0.59) | -0.09 (-0.42; 0.24) | -0.37 (-0.71;-0.03) | -0.78 (-1.20;-0.35) | -1.37 (-1.86;-0.87) | -2.19 (-2.73;-1.66) | -3.32 (-3.92;-2.72) | -4.80 (-5.65; -3.95) |
| GenR |  |  |  |  |  |  |  |  |
| Non-smoking | REF | REF | REF | REF | REF | REF | REF | REF |
| Quit smoking in early pregnancy | 0.06 (-0.56; 0.68) | 0.02 (-0.46; 0.49) | -0.01 (-0.57; 0.55) | 0.00 (-0.71; 0.70) | 0.05 (-0.75; 0.85) | 0.16 (-0.75; 1.07) | 0.34 (-0.89; 1.58) | 0.62 (-1.38; 2.62) |
| Continued smoking during pregnancy | 0.02 (-0.50; 0.55) | -0.26 (-0.65; 0.13) | -0.59 (-1.04;-0.14) | -0.99 (-1.55;-0.43) | -1.50 (-2.15;-0.86) | -2.15 (-2.88;-1.43) | -2.98 (-3.95;-2.00) | -4.01 (-5.57; -2.44) |
| BiB |  |  |  |  |  |  |  |  |
| Non-smoking | REF | REF | REF | REF | REF | REF | REF | REF |
| Quit smoking in early pregnancy | -0.45 (-1.86; 0.96) | -0.44 (-1.34; 0.45) | -0.46 (-1.18; 0.27) | -0.50 (-1.40; 0.40) | -0.59 (-1.69; 0.51) | -0.74 (-1.91; 0.42) | -0.97 (-2.10; 0.16) | -1.29 (-2.60; 0.03) |
| Continued smoking during pregnancy | 0.18 (-0.84; 1.20) | 0.07 (-0.59; 0.72) | -0.14 (-0.68; 0.40) | -0.52 (-1.18; 0.14) | -1.15 (-1.95;-0.35) | -2.10 (-2.94;-1.25) | -3.45 (-4.27;-2.62) | -5.28 (-6.26; -4.29) |
| **FL (mm)** | **12 wks** | **16 wks** | **20 wks** | **24 wks** | **28 wks** | **32 wks** | **36 wks** | **40 wks** |
| Maternal smoking during pregnancy |  |  |  |  |  |  |  |  |
| All |  |  |  |  |  |  |  |  |
| Non-smoking | REF | REF | REF | REF | REF | REF | REF | REF |
| Quit smoking in early pregnancy | -0.11 (-0.33; 0.12) | 0.01 (-0.12; 0.14) | 0.06 (-0.07; 0.19) | 0.05 (-0.10; 0.21) | -0.01 (-0.17; 0.15) | -0.14 (-0.33; 0.04) | -0.33 (-0.62;-0.05) | -0.59 (-1.05; -0.13) |
| Continued smoking during pregnancy | -0.16 (-0.35; 0.03) | -0.13 (-0.24;-0.03) | -0.17 (-0.27;-0.07) | -0.27 (-0.39;-0.15) | -0.43 (-0.55;-0.31) | -0.65 (-0.79;-0.50) | -0.93 (-1.14;-0.71) | -1.27 (-1.61; -0.92) |
| GenR |  |  |  |  |  |  |  |  |
| Non-smoking | REF | REF | REF | REF | REF | REF | REF | REF |
| Quit smoking in early pregnancy | -0.07 (-0.32; 0.19) | -0.02 (-0.19; 0.14) | 0.00 (-0.19; 0.19) | 0.01 (-0.20; 0.22) | -0.01 (-0.22; 0.20) | -0.05 (-0.31; 0.22) | -0.11 (-0.55; 0.34) | -0.18 (-0.91; 0.54) |
| Continued smoking during pregnancy | -0.26 (-0.48;-0.04) | -0.21 (-0.34;-0.07) | -0.23 (-0.38;-0.08) | -0.34 (-0.50;-0.17) | -0.52 (-0.69;-0.35) | -0.77 (-0.99;-0.56) | -1.11 (-1.47;-0.75) | -1.52 (-2.12; -0.92) |
| BiB |  |  |  |  |  |  |  |  |
| Non-smoking | REF | REF | REF | REF | REF | REF | REF | REF |
| Quit smoking in early pregnancy | -0.19 (-0.72; 0.34) | 0.00 (-0.26; 0.27) | 0.10 (-0.08; 0.29) | 0.11 (-0.12; 0.34) | 0.02 (-0.25; 0.28) | -0.17 (-0.45; 0.12) | -0.45 (-0.83;-0.07) | -0.82 (-1.44; -0.21) |
| Continued smoking during pregnancy | -0.02 (-0.42; 0.39) | -0.02 (-0.22; 0.18) | -0.07 (-0.21; 0.07) | -0.18 (-0.35; 0.00) | -0.33 (-0.52;-0.13) | -0.53 (-0.74;-0.33) | -0.79 (-1.06;-0.52) | -1.09 (-1.54; -0.64) |

**S7 Table. *Continued.***

|  | **Predicted mean difference (95% CI)** | | | | | | |
| --- | --- | --- | --- | --- | --- | --- | --- |
| **AC (mm)** | **16 wks** | **20 wks** | **24 wks** | **28 wks** | **32 wks** | **36 wks** | **40 wks** |
| Maternal smoking status during pregnancy |  |  |  |  |  |  |  |
| All |  |  |  |  |  |  |  |
| Non-smoking | REF | REF | REF | REF | REF | REF | REF |
| Quit smoking in early pregnancy | 0.04 (-0.83; 0.90) | -0.07 (-0.67; 0.54) | -0.07 (-0.86; 0.71) | 0.12 (-0.96; 1.20) | 0.59 (-0.60; 1.78) | 1.37 (-0.02; 2.77) | 2.51 (0.08; 4.93) |
| Continued smoking during pregnancy | 0.33 (-0.35; 1.01) | -0.08 (-0.55; 0.40) | -0.84 (-1.44;-0.23) | -1.86 (-2.69;-1.03) | -3.09 (-4.00; -2.19) | -4.50 (-5.55;-3.45) | -6.05 (-7.87; -4.23) |
| GenR |  |  |  |  |  |  |  |
| Non-smoking | REF | REF | REF | REF | REF | REF | REF |
| Quit smoking in early pregnancy | 0.92 (-1.58; 3.42) | 0.14 (-0.74; 1.02) | -0.40 (-2.18; 1.37) | -0.13 (-1.95; 1.69) | 1.30 (-0.80; 3.40) | 4.16 (-3.76; 12.07) | 8.63 (-9.30; 26.56) |
| Continued smoking during pregnancy | -0.04 (-2.08; 2.01) | -0.31 (-1.03; 0.41) | -0.89 (-2.30; 0.52) | -1.75 (-3.19;-0.31) | -2.87 (-4.58;-1.16) | -4.24 (-10.66;2.17) | -5.86 (-20.35; 8.64) |
| BiB |  |  |  |  |  |  |  |
| Non-smoking | REF | REF | REF | REF | REF | REF | REF |
| Quit smoking in early pregnancy | -0.07 (-1.40; 1.26) | -0.15 (-0.98; 0.69) | -0.12 (-1.45; 1.21) | 0.11 (-1.92; 2.14) | 0.59 (-1.68; 2.87) | 1.37 (-0.69; 3.44) | 2.48 (0.02; 4.94) |
| Continued smoking during pregnancy | 0.77 (-0.22; 1.75) | 0.14 (-0.50; 0.79) | -0.88 (-1.85; 0.09) | -2.10 (-3.55;-0.66) | -3.41 (-5.02;-1.81) | -4.72 (-6.19; -3.25) | -5.96 (-7.80; -4.12) |
| **EFW (g)** | **16 wks** | **20 wks** | **24 wks** | **28 wks** | **32 wks** | **36 wks** | **40 wks** |
| Maternal smoking status during pregnancy |  |  |  |  |  |  |  |
| All |  |  |  |  |  |  |  |
| Non-smoking | REF | REF | REF | REF | REF | REF | REF |
| Quit smoking in early pregnancy | -2.3 (-6.5; -2.0) | -1.7 (-4.9; 1.5) | -0.7 (-7.6; - 6.3) | 0.3 (-12.3; - 12.9) | 0.8 (-16.7; - 18.2) | 0.3 (-21.9; 22.5) | -1.6 (-33.5; 30.3) |
| Continued smoking during pregnancy | -1.4 (-4.7; 1.9) | -2.6 (-5.1; -0.1) | -9.8 (-15.1; -4.4) | -28.4 (-38.0;-18.7) | -63.8 (-77.1;-50.4) | -121.4 (-138.3;-104.4) | -206.6 (-231.3;-181.9) |
| GenR |  |  |  |  |  |  |  |
| Non-smoking | REF | REF | REF | REF | REF | REF | REF |
| Quit smoking in early pregnancy | 1.0 (-4.0; 6.1) | 1.0 (-3.0; 5.1) | 1.7 (-6.5; 10.0) | 3.9 (-11.1; 18.8) | 8.3 (-13.4; 29.9) | 15.8 (-13.8; 45.3) | 27.1 (-16.7; 70.9) |
| Continued smoking during pregnancy | -0.2 (-4.4; 4.0) | -3.2 (-6.5; 0.1) | -12.6 (-19.1; -6.0) | -31.0 (-43.0;-19.1) | -61.5 (-78.7;-44.2) | -106.7 (-130.3;-83.0) | -169.4 (-204.7;-134.1) |
| BiB |  |  |  |  |  |  |  |
| Non-smoking | REF | REF | REF | REF | REF | REF | REF |
| Quit smoking in early pregnancy | -6.5 (-14.2; 1.1) | -3.3 (-8.2; 1.6) | 1.4 (-12.0; 14.8) | 3.9 (-20.5; 28.4) | 0.8 (-31.0; 32.5) | -11.6 (-46.7; - 23.5) | -36.8 (-83.5; 9.9) |
| Continued smoking during pregnancy | -4.9 (-10.5; 0.6) | -1.2 (-5.0; 2.6) | -0.9 (-10.7; 8.8) | -14.6 (-32.1; - 3.0) | -52.6 (-75.3; -29.8) | -125.2 (-150.8;-99.6) | -242.9 (-278.0;-207.8) |

Predicted differences in mean head circumference (HC), femur length (FL), abdominal circumference (AC) and estimated fetal weight (EFW) comparing pre-pregnancy smokers who quit in early pregnancy and those continuing smoking during pregnancy with non-smokers (= reference category) at 4-weekly gestational age intervals from 12/16 weeks through 40 weeks. All mean differences (with 95% confidence intervals) are estimated using multilevel fractional polynomial models with adjustment for cohort, infant sex, maternal age, parity, height, body mass index, education and alcohol use during pregnancy.
