# Supplementary material for: Associations of maternal quitting, reducing, and continuing smoking during pregnancy with longitudinal fetal growth: Findings from Mendelian randomization and parental negative control studies
Source: PLoS Med. 2019 Nov 13;16(11):e1002972. doi: 10.1371/journal.pmed.1002972 (PMC6853297; doi:10.1371/journal.pmed.1002972)
Supplement: S8 Table — (DOCX) [file pmed.1002972.s020.docx]

**S8 Table. Maternal smoking intensity in continued smokers and predicted differences in mean fetal size (with 95% CIs) across gestation, overall and stratified by cohort.**

|  | **Predicted mean difference (95% CI)** | | | | | | | |
| --- | --- | --- | --- | --- | --- | --- | --- | --- |
| **HC (mm)** | **12 wks** | **16 wks** | **20 wks** | **24 wks** | **28 wks** | **32 wks** | **36 wks** | **40 wks** |
| Maternal smoking intensity |  |  |  |  |  |  |  |  |
| All |  |  |  |  |  |  |  |  |
| Non-smoker | REF | REF | REF | REF | REF | REF | REF | REF |
| Light smoker | 0.30 (-0.40; 1.01) | 0.07 (-0.44; 0.58) | -0.20 (-0.73; 0.34) | -0.54 (-1.21; 0.12) | -0.99 (-1.76;-0.23) | -1.58 (-2.39;-0.76) | -2.33 (-3.26;-1.40) | -3.29 (-4.62;-1.95) |
| Moderate smoker | 0.33 (-0.31; 0.96) | 0.10 (-0.35; 0.54) | -0.22 (-0.67; 0.24) | -0.67 (-1.24;-0.10) | -1.33 (-1.99;-0.67) | -2.26 (-2.96;-1.56) | -3.52 (-4.30;-2.75) | -5.18 (-6.28;-4.09) |
| Heavy smoker | -0.80 (-1.71; 0.12) | -0.86 (-1.48;-0.24) | -1.03 (-1.63;-0.43) | -1.38 (-2.13;-0.63) | -1.98 (-2.86;-1.10) | -2.92 (-3.85;-1.99) | -4.28 (-5.32;-3.24) | -6.13 (-7.63;-4.63) |
| GenR |  |  |  |  |  |  |  |  |
| Non-smoker | REF | REF | REF | REF | REF | REF | REF | REF |
| Light smoker | 0.19 (-0.56; 0.94) | 0.00 (-0.56; 0.56) | -0.22 (-0.86; 0.42) | -0.51 (-1.31; 0.30) | -0.88 (-1.81; 0.04) | -1.38 (-2.41;-0.35) | -2.02 (-3.39;-0.66) | -2.84 (-5.02;-0.66) |
| Moderate smoker | 0.25 (-0.49; 0.99) | -0.15 (-0.71; 0.42) | -0.57 (-1.23; 0.09) | -1.05 (-1.88;-0.22) | -1.62 (-2.56;-0.67) | -2.28 (-3.35;-1.22) | -3.08 (-4.50;-1.66) | -4.03 (-6.30;-1.76) |
| Heavy smoker | -0.76 (-1.88; 0.37) | -1.01 (-1.81;-0.22) | -1.35 (-2.22;-0.47) | -1.82 (-2.93;-0.71) | -2.50 (-3.77;-1.22) | -3.43 (-4.88;-1.98) | -4.69 (-6.69;-2.68) | -6.32 (-9.62;-3.03) |
| BiB |  |  |  |  |  |  |  |  |
| Non-smoker | REF | REF | REF | REF | REF | REF | REF | REF |
| Light smoker | 0.58 (-1.20; 2.35) | 0.26 (-0.88; 1.41) | -0.09 (-1.02; 0.83) | -0.54 (-1.65; 0.58) | -1.10 (-2.44; 0.25) | -1.81 (-3.23;-0.39) | -2.72 (-4.11;-1.33) | -3.86 (-5.53;-2.20) |
| Moderate smoker | 0.38 (-0.89; 1.65) | 0.28 (-0.54; 1.09) | 0.06 (-0.60; 0.72) | -0.34 (-1.15; 0.46) | -1.03 (-1.99;-0.06) | -2.08 (-3.09;-1.06) | -3.58 (-4.57;-2.58) | -5.61 (-6.81;-4.40) |
| Heavy smoker | -1.52 (-3.20; 0.16) | -1.10 (-2.19;-0.01) | -0.84 (-1.71; 0.02) | -0.86 (-1.90; 0.17) | -1.29 (-2.53;-0.04) | -2.24 (-3.56;-0.93) | -3.85 (-5.15;-2.56) | -6.24 (-7.84;-4.64) |
| **FL (mm)** | **12 wks** | **16 wks** | **20 wks** | **24 wks** | **28 wks** | **32 wks** | **36 wks** | **40 wks** |
| Maternal smoking intensity |  |  |  |  |  |  |  |  |
| All |  |  |  |  |  |  |  |  |
| Non-smoker | REF | REF | REF | REF | REF | REF | REF | REF |
| Light smoker | -0.16 (-0.45; 0.13) | -0.14 (-0.31; 0.02) | -0.17 (-0.33;-0.01) | -0.24 (-0.42;-0.05) | -0.35 (-0.53;-0.16) | -0.50 (-0.72;-0.28) | -0.69 (-1.04;-0.35) | -0.93 (-1.50;-0.36) |
| Moderate smoker | -0.14 (-0.39; 0.11) | -0.10 (-0.24; 0.04) | -0.13 (-0.26; 0.00) | -0.23 (-0.39;-0.08) | -0.40 (-0.57;-0.24) | -0.65 (-0.84;-0.46) | -0.96 (-1.24;-0.69) | -1.35 (-1.79;-0.91) |
| Heavy smoker | -0.18 (-0.55; 0.18) | -0.22 (-0.42;-0.02) | -0.30 (-0.48;-0.13) | -0.44 (-0.64;-0.24) | -0.63 (-0.85;-0.41) | -0.87 (-1.11;-0.63) | -1.16 (-1.52;-0.81) | -1.51 (-2.08;-0.93) |
| GenR |  |  |  |  |  |  |  |  |
| Non-smoker | REF | REF | REF | REF | REF | REF | REF | REF |
| Light smoker | -0.28 (-0.61; 0.05) | -0.24 (-0.44;-0.04) | -0.25 (-0.46;-0.03) | -0.30 (-0.54;-0.06) | -0.39 (-0.63;-0.15) | -0.54 (-0.84;-0.23) | -0.72 (-1.25;-0.20) | -0.96 (-1.83;-0.08) |
| Moderate smoker | -0.23 (-0.54; 0.08) | -0.12 (-0.32; 0.07) | -0.12 (-0.35; 0.10) | -0.22 (-0.47; 0.02) | -0.43 (-0.68;-0.18) | -0.75 (-1.07;-0.43) | -1.16 (-1.70;-0.62) | -1.69 (-2.58;-0.79) |
| Heavy smoker | -0.29 (-0.76; 0.18) | -0.32 (-0.60;-0.05) | -0.43 (-0.73;-0.14) | -0.63 (-0.96;-0.30) | -0.91 (-1.25;-0.58) | -1.28 (-1.70;-0.85) | -1.73 (-2.45;-1.00) | -2.26 (-3.47;-1.05) |
| BiB |  |  |  |  |  |  |  |  |
| Non-smoker | REF | REF | REF | REF | REF | REF | REF | REF |
| Light smoker | 0.24 (-0.46; 0.95) | 0.10 (-0.25; 0.45) | -0.05 (-0.29; 0.19) | -0.19 (-0.49; 0.10) | -0.34 (-0.67; 0.00) | -0.48 (-0.83;-0.13) | -0.61 (-1.09;-0.14) | -0.75 (-1.54; 0.03) |
| Moderate smoker | 0.07 (-0.43; 0.57) | 0.02 (-0.23; 0.27) | -0.07 (-0.24; 0.10) | -0.20 (-0.41; 0.01) | -0.37 (-0.61;-0.13) | -0.58 (-0.83;-0.33) | -0.83 (-1.16;-0.50) | -1.12 (-1.67;-0.58) |
| Heavy smoker | -0.40 (-1.06; 0.26) | -0.24 (-0.58; 0.09) | -0.18 (-0.41; 0.04) | -0.22 (-0.49; 0.05) | -0.36 (-0.66;-0.05) | -0.59 (-0.91;-0.27) | -0.92 (-1.34;-0.50) | -1.35 (-2.04;-0.66) |

**S8 Table. *Continued.***

| **AC (mm)** | **16 wks** | **20 wks** | **24 wks** | **28 wks** | **32 wks** | **36 wks** | **40 wks** |
| --- | --- | --- | --- | --- | --- | --- | --- |
| Maternal smoking intensity |  |  |  |  |  |  |  |
| All |  |  |  |  |  |  |  |
| Non-smoker | REF | REF | REF | REF | REF | REF | REF |
| Light smoker | 0.53 (-0.53; 1.59) | -0.02 (-0.77; 0.74) | -0.78 (-1.71; 0.15) | -1.52 (-2.76;-0.27) | -2.09 (-3.45;-0.72) | -2.38 (-4.07;-0.69) | -2.32 (-5.37; 0.72) |
| Moderate smoker | 0.55 (-0.34; 1.44) | 0.01 (-0.62; 0.64) | -0.94 (-1.75;-0.14) | -2.17 (-3.29;-1.06) | -3.59 (-4.81;-2.37) | -5.14 (-6.48;-3.80) | -6.78 (-9.00; -4.56) |
| Heavy smoker | -0.16 (-1.32; 1.00) | -0.40 (-1.21; 0.42) | -1.04 (-2.08; 0.00) | -2.13 (-3.56;-0.69) | -3.68 (-5.26;-2.10) | -5.72 (-7.49;-3.95) | -8.25 (-11.19; -5.31) |
| GenR |  |  |  |  |  |  |  |
| Non-smoker | REF | REF | REF | REF | REF | REF | REF |
| Light smoker | 0.71 (-2.07; 3.50) | -0.09 (-1.11; 0.92) | -0.98 (-2.98; 1.03) | -1.46 (-3.50; 0.57) | -1.28 (-3.69; 1.13) | -0.23 (-9.21; 8.75) | 1.85 (-18.41; 22.12) |
| Moderate smoker | -2.12 (-5.34; 1.10) | -0.65 (-1.72; 0.41) | 0.11 (-2.09; 2.32) | -1.05 (-3.26; 1.16) | -4.88 (-7.57;-2.19) | -11.90 (-22.26;-1.54) | -22.52 (-45.92; 0.87) |
| Heavy smoker | 2.17 (-1.60; 5.94) | -0.09 (-1.50; 1.31) | -2.50 (-5.10; 0.10) | -3.71 (-6.44;-0.98) | -2.93 (-6.08; 0.22) | 0.40 (-11.07; 11.87) | 6.74 (-19.22; 32.70) |
| BiB |  |  |  |  |  |  |  |
| Non-smoker | REF | REF | REF | REF | REF | REF | REF |
| Light smoker | 1.28 (-0.43; 2.98) | 0.22 (-0.91; 1.36) | -1.15 (-2.78; 0.48) | -2.35 (-4.77; 0.07) | -3.08 (-5.77;-0.39) | -3.12 (-5.60; -0.64) | -2.31 (-5.41; 0.78) |
| Moderate smoker | 1.10 (-0.09; 2.29) | 0.32 (-0.47; 1.10) | -0.94 (-2.10; 0.23) | -2.39 (-4.13;-0.66) | -3.90 (-5.82;-1.97) | -5.34 (-7.11; -3.57) | -6.63 (-8.86; -4.39) |
| Heavy smoker | -0.37 (-1.88; 1.13) | -0.40 (-1.40; 0.60) | -0.80 (-2.26; 0.66) | -1.71 (-3.87; 0.46) | -3.21 (-5.62;-0.80) | -5.39 (-7.66; -3.11) | -8.27 (-11.24; -5.30) |
| **EFW (g)** | **16 wks** | **20 wks** | **24 wks** | **28 wks** | **32 wks** | **36 wks** | **40 wks** |
| Maternal smoking during pregnancy |  |  |  |  |  |  |  |
| All |  |  |  |  |  |  |  |
| Non-smoker | REF | REF | REF | REF | REF | REF | REF |
| Light smoker | -1.3 (-6.5; - 3.8) | -3.1 (-7.0; 0.9) | -9.5 (-17.6; -1.5) | -23.8 (-38.4; -9.2) | -49.0 (-69.3; -28.6) | -87.9 (-114.3, -61.6) | -143.8 (-182.2; -105.5) |
| Moderate smoker | -0.7 (-5.1; 3.7) | -1.6 (-4.9; 1.8) | -8.4 (-15.7; -1.2) | -27.3 (-40.3;-14.2) | -64.0 (-81.8; -46.1) | -124.5 (-147.0; -102.0) | -214.8 (-247.6;-182.1) |
| Heavy smoker | -5.9 (-11.7; -0.1) | -5.8 (-10.0; -1.5) | -12.6 (-22.0; -3.3) | -35.6 (-52.5;-18.7) | -83.5 (-106.7; -60.3) | -165.4 (-194.9; -135.9) | -290.1 (-333.6; -246.7) |
| GenR |  |  |  |  |  |  |  |
| Non-smoker | REF | REF | REF | REF | REF | REF | REF |
| Light smoker | 0.5 (-5.4; 6.3) | -2.1 (-6.8; 2.6) | -9.3 (-18.6; 0.1) | -22.3 (-39.3; -5.3) | -42.5 (-67.2; -17.9) | -71.3 (-105.1; -37.5) | -110.0 (-160.1; -59.9) |
| Moderate smoker | -0.9 (-7.1; 5.3) | -3.0 (-7.8; 1.9) | -11.1 (-20.8; -1.4) | -29.3 (-47.1,-11.6) | -61.5 (-87.3; -35.7) | -111.8 (-147.2; -76.4) | -184.0 (-236.9; -131.2) |
| Heavy smoker | 0.2 (-8.1; 8.6) | -5.5 (-11.9; 1.0) | -217 (-34.5; -8.8) | -52.0 (-75.5;-28.6) | -100.1 (-134.1;-66.1) | -169.5 (-216.4; -122.7) | -263.9 (-334.8; -192.9) |
| BiB |  |  |  |  |  |  |  |
| Non-smoker | REF | REF | REF | REF | REF | REF | REF |
| Light smoker | -5.8 (-15.5; 4.0) | -3.53 (-10.2; 3.2) | -4.5 (-20.8; 11.9) | -16.1 (-45.5; 13.3) | -45.9 (-84.2; -7.7) | -101.6 (-144.6; -58.5) | -190.5 (-250.0; -131.0) |
| Moderate smoker | -3.0 (-9.8; 3.8) | -0.03 (-4.7; 4.6) | -1.00 (-12.8; 10.8) | -15.6 (-36.8; 5.6) | -53.6 (-81.0; -26.1) | -124.6 (-155.4; -93.8) | -238.4 (-281.0; -195.7) |
| Heavy smoker | -11.4 (-20.0; -2.8) | -4.5 (-10.4; 1.4) | -0.7 (-15.6; 14.2) | -14.8 (-41.6; 12.0) | -61.7 (-96.7; -26.8) | -156.3 (-196.2; -116.5) | -313.5 (-369.4; -257.6) |

Predicted differences in mean head circumference (HC), femur length (FL), abdominal circumference (AC) and estimated fetal weight (EFW) comparing different categories of smoking quantity in pre-pregnancy smokers continuing smoking during pregnancy with non-smokers (= reference category) at 4-weekly gestational age intervals from 12/16 weeks through 40 weeks. All mean differences (with 95% confidence intervals) are estimated using multilevel fractional polynomial models with adjustment for cohort, infant sex, maternal age, parity, height, body mass index, education and alcohol use during pregnancy.
