# Supplementary material for: Associations of maternal quitting, reducing, and continuing smoking during pregnancy with longitudinal fetal growth: Findings from Mendelian randomization and parental negative control studies
Source: PLoS Med. 2019 Nov 13;16(11):e1002972. doi: 10.1371/journal.pmed.1002972 (PMC6853297; doi:10.1371/journal.pmed.1002972)
Supplement: S9 Table — (DOCX) [file pmed.1002972.s021.docx]

**S9 Table. Maternal smoking during pregnancy and predicted differences in mean fetal size across gestation as a proportion of the mean, overall and stratified by cohort.**

|  | **Predicted mean difference as a proportion of the mean** | | | | | | | |
| --- | --- | --- | --- | --- | --- | --- | --- | --- |
| **HC (mm)** | **12 wks** | **16 wks** | **20 wks** | **24 wks** | **28 wks** | **32 wks** | **36 wks** | **40 wks** |
| Maternal smoking during pregnancy |  |  |  |  |  |  |  |  |
| All |  |  |  |  |  |  |  |  |
| Non-smoking | REF | REF | REF | REF | REF | REF | REF | REF |
| Quit smoking in early pregnancy | -0.0015 | -0.0014 | -0.0014 | -0.0014 | -0.0013 | -0.0013 | -0.0013 | -0.0013 |
| Continued smoking during pregnancy | 0.0016 | -0.0007 | -0.0023 | -0.0039 | -0.0059 | -0.0085 | -0.0120 | -0.0170 |
| GenR |  |  |  |  |  |  |  |  |
| Non-smoking | REF | REF | REF | REF | REF | REF | REF | REF |
| Quit smoking in early pregnancy | 0.0009 | 0.0002 | -0.0001 | 0.0000 | 0.0002 | 0.0006 | 0.0013 | 0.0023 |
| Continued smoking during pregnancy | 0.0003 | -0.0023 | -0.0037 | -0.0050 | -0.0065 | -0.0085 | -0.0111 | -0.0149 |
| BiB |  |  |  |  |  |  |  |  |
| Non-smoking | REF | REF | REF | REF | REF | REF | REF | REF |
| Quit smoking in early pregnancy | -0.0051 | -0.0035 | -0.0028 | -0.0025 | -0.0026 | -0.0029 | -0.0035 | -0.0044 |
| Continued smoking during pregnancy | 0.0020 | 0.0005 | -0.0009 | -0.0026 | -0.0050 | -0.0082 | -0.0125 | -0.0181 |
| **FL (mm)** | **12 wks** | **16 wks** | **20 wks** | **24 wks** | **28 wks** | **32 wks** | **36 wks** | **40 wks** |
| Maternal smoking during pregnancy |  |  |  |  |  |  |  |  |
| All |  |  |  |  |  |  |  |  |
| Non-smoking | REF | REF | REF | REF | REF | REF | REF | REF |
| Quit smoking in early pregnancy | -0.0157 | 0.0004 | 0.0023 | 0.0016 | -0.0003 | -0.0030 | -0.0063 | -0.0104 |
| Continued smoking during pregnancy | -0.0231 | -0.0078 | -0.0064 | -0.0077 | -0.0102 | -0.0135 | -0.0175 | -0.0224 |
| GenR |  |  |  |  |  |  |  |  |
| Non-smoking | REF | REF | REF | REF | REF | REF | REF | REF |
| Quit smoking in early pregnancy | -0.0108 | -0.0012 | 0.0001 | 0.0002 | -0.0002 | -0.0010 | -0.0021 | -0.0035 |
| Continued smoking during pregnancy | -0.0421 | -0.0118 | -0.0085 | -0.0095 | -0.0123 | -0.0164 | -0.0219 | -0.0289 |
| BiB |  |  |  |  |  |  |  |  |
| Non-smoking | REF | REF | REF | REF | REF | REF | REF | REF |
| Quit smoking in early pregnancy | -0.0333 | 0.0002 | 0.0040 | 0.0032 | 0.0005 | -0.0034 | -0.0083 | -0.0142 |
| Continued smoking during pregnancy | -0.0030 | -0.0012 | -0.0028 | -0.0050 | -0.0078 | -0.0109 | -0.0146 | -0.0188 |

**S9 Table. *Continued.***

|  | **Predicted mean difference as a proportion of the mean** | | | | | | |
| --- | --- | --- | --- | --- | --- | --- | --- |
| **AC (mm)** | **16 wks** | **20 wks** | **24 wks** | **28 wks** | **32 wks** | **36 wks** | **40 wks** |
| Maternal smoking status during pregnancy |  |  |  |  |  |  |  |
| All |  |  |  |  |  |  |  |
| Non-smoking | REF | REF | REF | REF | REF | REF | REF |
| Quit smoking in early pregnancy | 0.0003 | -0.0005 | -0.0004 | 0.0006 | 0.0025 | 0.0054 | 0.0096 |
| Continued smoking during pregnancy | 0.0030 | -0.0006 | -0.0049 | -0.0090 | -0.0130 | -0.0176 | -0.0232 |
| GenR |  |  |  |  |  |  |  |
| Non-smoking | REF | REF | REF | REF | REF | REF | REF |
| Quit smoking in early pregnancy | 0.0082 | 0.0011 | -0.0024 | -0.0007 | 0.0053 | 0.0148 | 0.0277 |
| Continued smoking during pregnancy | -0.0003 | -0.0023 | -0.0053 | -0.0085 | -0.0117 | -0.0151 | -0.0188 |
| BiB |  |  |  |  |  |  |  |
| Non-smoking | REF | REF | REF | REF | REF | REF | REF |
| Quit smoking in early pregnancy | -0.0007 | -0.0011 | -0.0007 | 0.0005 | 0.0025 | 0.0054 | 0.0095 |
| Continued smoking during pregnancy | 0.0068 | 0.0011 | -0.0051 | -0.0102 | -0.0144 | -0.0185 | -0.0229 |
| **EFW (g)** | **16 wks** | **20 wks** | **24 wks** | **28 wks** | **32 wks** | **36 wks** | **40 wks** |
| Maternal smoking status during pregnancy |  |  |  |  |  |  |  |
| All |  |  |  |  |  |  |  |
| Non-smoking | REF | REF | REF | REF | REF | REF | REF |
| Quit smoking in early pregnancy | -0.0154 | -0.0062 | -0.0014 | 0.0004 | 0.0008 | 0.0003 | -0.0018 |
| Continued smoking during pregnancy | -0.0096 | -0.0096 | -0.0196 | -0.0377 | -0.0665 | -0.1166 | -0.2237 |
| GenR |  |  |  |  |  |  |  |
| Non-smoking | REF | REF | REF | REF | REF | REF | REF |
| Quit smoking in early pregnancy | 0.0079 | 0.0044 | 0.0039 | 0.0059 | 0.0099 | 0.0173 | 0.0333 |
| Continued smoking during pregnancy | -0.0014 | -0.0135 | -0.0289 | -0.0473 | -0.0735 | -0.1171 | -0.2080 |
| BiB |  |  |  |  |  |  |  |
| Non-smoking | REF | REF | REF | REF | REF | REF | REF |
| Quit smoking in early pregnancy | -0.0481 | -0.0116 | 0.0025 | 0.0048 | 0.0008 | -0.0125 | -0.0632 |
| Continued smoking during pregnancy | -0.0364 | -0.0042 | -0.0017 | -0.0181 | -0.0540 | -0.1341 | -0.4170 |

Predicted differences in mean head circumference (HC), femur length (FL), abdominal circumference (AC) and estimated fetal weight (EFW) as a proportion of the mean comparing pre-pregnancy smokers who quit early in pregnancy and pre-pregnancy smokers continuing smoking through pregnancy with non-smokers (= reference category) at 4-weekly gestational age intervals from 12/16 weeks through 40 weeks. All proportional differences in mean fetal size are estimated using multilevel fractional polynomial models with adjustment for cohort, infant sex, maternal age, parity, height, body mass index, education and alcohol use during pregnancy.
