# Supplementary material for: Associations of maternal quitting, reducing, and continuing smoking during pregnancy with longitudinal fetal growth: Findings from Mendelian randomization and parental negative control studies
Source: PLoS Med. 2019 Nov 13;16(11):e1002972. doi: 10.1371/journal.pmed.1002972 (PMC6853297; doi:10.1371/journal.pmed.1002972)
Supplement: S10 Table — (DOCX) [file pmed.1002972.s022.docx]

**S10 Table. Maternal smoking intensity in continued smokers and predicted differences in mean fetal size across gestation as a proportion of the mean, overall and stratified by cohort.**

|  | **Predicted mean difference as a proportion of the mean** | | | | | | | |
| --- | --- | --- | --- | --- | --- | --- | --- | --- |
| **HC (mm)** | **12 wks** | **16 wks** | **20 wks** | **24 wks** | **28 wks** | **32 wks** | **36 wks** | **40 wks** |
| Maternal smoking intensity |  |  |  |  |  |  |  |  |
| All |  |  |  |  |  |  |  |  |
| Non-smoker | REF | REF | REF | REF | REF | REF | REF | REF |
| Light smoker | 0.0039 | 0.0006 | -0.0012 | -0.0027 | -0.0042 | -0.0061 | -0.0084 | -0.0116 |
| Moderate smoker | 0.0042 | 0.0008 | -0.0013 | -0.0033 | -0.0057 | -0.0087 | -0.0128 | -0.0183 |
| Heavy smoker | -0.0102 | -0.0071 | -0.0063 | -0.0068 | -0.0085 | -0.0113 | -0.0155 | -0.0217 |
| GenR |  |  |  |  |  |  |  |  |
| Non-smoker | REF | REF | REF | REF | REF | REF | REF | REF |
| Light smoker | 0.0027 | 0.0000 | -0.0014 | -0.0025 | -0.0038 | -0.0054 | -0.0075 | -0.0106 |
| Moderate smoker | 0.0036 | -0.0013 | -0.0036 | -0.0053 | -0.0070 | -0.0089 | -0.0115 | -0.0150 |
| Heavy smoker | -0.0108 | -0.0087 | -0.0084 | -0.0091 | -0.0108 | -0.0134 | -0.0174 | -0.0235 |
| BiB |  |  |  |  |  |  |  |  |
| Non-smoker | REF | REF | REF | REF | REF | REF | REF | REF |
| Light smoker | 0.0066 | 0.0021 | -0.0006 | -0.0027 | -0.0048 | -0.0071 | -0.0098 | -0.0133 |
| Moderate smoker | 0.0044 | 0.0022 | 0.0004 | -0.0017 | -0.0045 | -0.0081 | -0.0129 | -0.0193 |
| Heavy smoker | -0.0173 | -0.0087 | -0.0051 | -0.0043 | -0.0056 | -0.0088 | -0.0139 | -0.0215 |
| **FL (mm)** | **12 wks** | **16 wks** | **20 wks** | **24 wks** | **28 wks** | **32 wks** | **36 wks** | **40 wks** |
| Maternal smoking intensity |  |  |  |  |  |  |  |  |
| All |  |  |  |  |  |  |  |  |
| Non-smoker | REF | REF | REF | REF | REF | REF | REF | REF |
| Light smoker | -0.0231 | -0.0081 | -0.0062 | -0.0067 | -0.0082 | -0.0104 | -0.0132 | -0.0166 |
| Moderate smoker | -0.0203 | -0.0057 | -0.0048 | -0.0066 | -0.0096 | -0.0135 | -0.0183 | -0.0241 |
| Heavy smoker | -0.0262 | -0.0123 | -0.0112 | -0.0125 | -0.0149 | -0.0181 | -0.0221 | -0.0269 |
| GenR |  |  |  |  |  |  |  |  |
| Non-smoker | REF | REF | REF | REF | REF | REF | REF | REF |
| Light smoker | -0.0458 | -0.0136 | -0.0089 | -0.0083 | -0.0093 | -0.0113 | -0.0142 | -0.0181 |
| Moderate smoker | -0.0378 | -0.0071 | -0.0044 | -0.0063 | -0.0102 | -0.0156 | -0.0228 | -0.0318 |
| Heavy smoker | -0.0472 | -0.0181 | -0.0157 | -0.0176 | -0.0214 | -0.0268 | -0.0338 | -0.0427 |
| BiB |  |  |  |  |  |  |  |  |
| Non-smoker | REF | REF | REF | REF | REF | REF | REF | REF |
| Light smoker | 0.0370 | 0.0056 | -0.0019 | -0.0055 | -0.0080 | -0.0099 | -0.0116 | -0.0132 |
| Moderate smoker | 0.0100 | 0.0010 | -0.0027 | -0.0058 | -0.0089 | -0.0121 | -0.0157 | -0.0197 |
| Heavy smoker | -0.0608 | -0.0142 | -0.0069 | -0.0064 | -0.0085 | -0.0123 | -0.0173 | -0.0237 |

**S10 Table. *Continued.***

|  | **Predicted mean difference as a proportion of the mean** | | | | | | |
| --- | --- | --- | --- | --- | --- | --- | --- |
|  | **12 wks** | **12 wks** | **12 wks** | **12 wks** | **12 wks** | **12 wks** | **12 wks** |
| Maternal smoking intensity |  |  |  |  |  |  |  |
| All |  |  |  |  |  |  |  |
| Non-smoker | REF | REF | REF | REF | REF | REF | REF |
| Light smoker | 0.0048 | -0.0001 | -0.0045 | -0.0073 | -0.0087 | -0.0092 | -0.0088 |
| Moderate smoker | 0.0049 | 0.0001 | -0.0055 | -0.0104 | -0.0151 | -0.0199 | -0.0257 |
| Heavy smoker | -0.0014 | -0.0029 | -0.0060 | -0.0102 | -0.0154 | -0.0221 | -0.0312 |
| GenR |  |  |  |  |  |  |  |
| Non-smoker | REF | REF | REF | REF | REF | REF | REF |
| Light smoker | 0.0060 | -0.0007 | -0.0058 | -0.0071 | -0.0052 | -0.0008 | 0.0055 |
| Moderate smoker | -0.0179 | -0.0048 | 0.0007 | -0.0051 | -0.0197 | -0.0406 | -0.0664 |
| Heavy smoker | 0.0183 | -0.0007 | -0.0150 | -0.0181 | -0.0118 | 0.0014 | 0.0199 |
| BiB |  |  |  |  |  |  |  |
| Non-smoker | REF | REF | REF | REF | REF | REF | REF |
| Light smoker | 0.0112 | 0.0016 | -0.0067 | -0.0114 | -0.0131 | -0.0122 | -0.0088 |
| Moderate smoker | 0.0096 | 0.0023 | -0.0054 | -0.0116 | -0.0165 | -0.0209 | -0.0253 |
| Heavy smoker | -0.0033 | -0.0029 | -0.0046 | -0.0083 | -0.0136 | -0.0211 | -0.0316 |
| **EFW (g)** | **16 wks** | **20 wks** | **24 wks** | **28 wks** | **32 wks** | **36 wks** | **40 wks** |
| Maternal smoking during pregnancy |  |  |  |  |  |  |  |
| All |  |  |  |  |  |  |  |
| Non-smoker | REF | REF | REF | REF | REF | REF | REF |
| Light smoker | -0.0089 | -0.0110 | -0.0188 | -0.0315 | -0.0520 | -0.0896 | -0.1817 |
| Moderate smoker | -0.0046 | -0.0056 | -0.0166 | -0.0361 | -0.0679 | -0.1269 | -0.2714 |
| Heavy smoker | -0.0397 | -0.0207 | -0.0250 | -0.0471 | -0.0887 | -0.1686 | -0.3666 |
| GenR |  |  |  |  |  |  |  |
| Non-smoker | REF | REF | REF | REF | REF | REF | REF |
| Light smoker | 0.0035 | -0.0086 | -0.0203 | -0.0324 | -0.0486 | -0.0753 | -0.1320 |
| Moderate smoker | -0.0068 | -0.0121 | -0.0244 | -0.0426 | -0.0703 | -0.1180 | -0.2209 |
| Heavy smoker | 0.0017 | -0.0221 | -0.0476 | -0.0756 | -0.1143 | -0.1790 | -0.3167 |
| BiB |  |  |  |  |  |  |  |
| Non-smoker | REF | REF | REF | REF | REF | REF | REF |
| Light smoker | -0.0412 | -0.0122 | -0.0080 | -0.0195 | -0.0458 | -0.1035 | -0.2910 |
| Moderate smoker | -0.0216 | -0.0001 | -0.0018 | -0.0189 | -0.0534 | -0.1270 | -0.3641 |
| Heavy smoker | -0.0810 | -0.0155 | -0.0013 | -0.0179 | -0.0615 | -0.1594 | -0.4789 |

Predicted differences in mean head circumference (HC), femur length (FL), abdominal circumference (AC) and estimated fetal weight (EFW) as a proportion of the mean comparing different categories of smoking quantity in pre-pregnancy smokers continuing smoking through pregnancy with non-smokers (= reference category) at 4-weekly gestational age intervals from 12/16 weeks through 40 weeks. All proportional differences in mean fetal size are estimated using multilevel fractional polynomial models with adjustment for cohort, infant sex, maternal age, parity, height, body mass index, education and alcohol use during pregnancy.
