# Supplementary material for: Associations of maternal quitting, reducing, and continuing smoking during pregnancy with longitudinal fetal growth: Findings from Mendelian randomization and parental negative control studies
Source: PLoS Med. 2019 Nov 13;16(11):e1002972. doi: 10.1371/journal.pmed.1002972 (PMC6853297; doi:10.1371/journal.pmed.1002972)
Supplement: S11 Table — (DOCX) [file pmed.1002972.s023.docx]

**S11 Table. Association of maternal rs10151730 genotype with maternal smoking variables.**

|  | **Continued smoking during pregnancy in pre-pregnancy smokers** | | |
| --- | --- | --- | --- |
|  | N continued smoking  no / yes | Risk allele frequency  continued smoking  no / yes | OR (95% CI) per T allele increase |
| GenR | 332 / 650 | 0.32 / 0.36 | 1.17 (0.96; 1.43) |
| BiB | 356 / 892 | 0.31 / 0.32 | 1.03 (0.86; 1.24) |
| All | 688 / 1542 | 0.31 / 0.33 | 1.10 (0.96; 1.25) |
|  | **Smoking intensity in pre-pregnancy smokers quitting smoking in early pregnancy** | | |
|  | N heavy smoking  no / yes | Risk allele frequency  heavy smoking  no / yes | OR (95% CI) per T allele increase |
| GenR | 249 / 80 | 0.33 / 0.30 | 0.88 (0.61; 1.28) |
| BiB | 276 / 80 | 0.30 / 0.34 | 1.19 (0.82; 1.72) |
| All | 525/ 160 | 0.31 / 0.32 | 1.02 (0.79; 1.33) |
|  | **Smoking intensity in pre-pregnancy smokers continuing smoking during pregnancy** | | |
|  | N heavy smoking  no / yes | Risk allele frequency  heavy smoking  no / yes | OR (95% CI) per T allele increase |
| GenR | 517 / 133 | 0.35 / 0.39 | 1.18 (0.89; 1.57) |
| BiB | 647 / 245 | 0.30 / 0.36 | 1.27 (1.03; 1.58) |
| All | 1164 / 378 | 0.32 / 0.37 | 1.24 (1.04; 1.47) |
|  | **Pre-pregnancy smoking** | | |
|  | N  no / yes | Risk allele frequency  heavy smoking  no / yes | OR (95% CI) per T allele increase |
| GenR | 2622 / 982 | 0.33 / 0.34 | 1.08 (0.97; 1.20) |
| BiB | 1675 / 1248 | 0.33 / 0.31 | 0.91 (0.82-1.02) |
| All | 4297 / 2230 | 0.33 / 0.33 | 1.00 (0.92-1.08) |

Association of maternal rs1051730 genotype with 1). smoking cessation among pre-pregnancy smokers; 2). smoking intensity in pre-pregnancy smokers quitting and continuing smoking during pregnancy and 3). likelihood of being a pre-pregnancy smoker in the total analysis cohort with genotyping data. Odds ratios for continued smoking during pregnancy (yes vs. no) and heavy smoking, approximately > 10 cigarettes per day (yes vs. no) per maternal rs1051730 T allele increase, overall and stratified by cohort. Abbreviations: OR = odds ratio; CI = confidence interval.
