# Supplementary material for: Associations of maternal quitting, reducing, and continuing smoking during pregnancy with longitudinal fetal growth: Findings from Mendelian randomization and parental negative control studies
Source: PLoS Med. 2019 Nov 13;16(11):e1002972. doi: 10.1371/journal.pmed.1002972 (PMC6853297; doi:10.1371/journal.pmed.1002972)
Supplement: S12 Table — (DOCX) [file pmed.1002972.s024.docx]

**S12 Table. Predicted differences in mean fetal size (with 95% CIs) across gestation per risk allele increase at rs1051730 in non-smokers, pre-pregnancy smokers who quit in early pregnancy and pre-pregnancy smokers continuing smoking through pregnancy, overall and stratified by cohort.**

|  | **Predicted mean difference (95% CI)** | | | | | | | |
| --- | --- | --- | --- | --- | --- | --- | --- | --- |
| **HC (mm)** | **12 wks** | **16 wks** | **20 wks** | **24 wks** | **28 wks** | **32 wks** | **36 wks** | **40 wks** |
| Rs1051730, per risk allele increase |  |  |  |  |  |  |  |  |
| All |  |  |  |  |  |  |  |  |
| Non-smoking | -0.19 (-0.50; 0.13) | -0.11(-0.35; 0.13) | -0.04(-0.30; 0.23) | 0.04(-0.30; 0.38) | 0.12(-0.27; 0.51) | 0.20(-0.22; 0.63) | 0.29(-0.21; 0.79) | 0.38(-0.34; 1.10) |
| Quit smoking in early pregnancy | 0.44 (-0.38; 1.26) | 0.39(-0.19; 0.98) | 0.31(-0.35; 0.97) | 0.17(-0.70; 1.04) | -0.06(-1.09; 0.98) | -0.40(-1.52; 0.73) | -0.88(-2.14; 0.39) | -1.52(-3.24; 0.20) |
| Continued smoking during pregnancy | -0.66(-1.30; -0.02) | -0.42(-0.89; 0.04) | -0.23(-0.70; 0.24) | -0.12(-0.70; 0.47) | -0.11(-0.79; 0.56) | -0.26(-0.98; 0.47) | -0.58(-1.39; 0.23) | -1.11(-2.24; 0.01) |
| GenR |  |  |  |  |  |  |  |  |
| Non-smoking | -0.21(-0.54; 0.12) | -0.23(-0.48; 0.02) | -0.22(-0.52; 0.08) | -0.17(-0.56; 0.22) | -0.07(-0.52; 0.38) | 0.11(-0.40; 0.62) | 0.38(-0.30; 1.06) | 0.75(-0.32; 1.82) |
| Quit smoking in early pregnancy | 0.54(-0.39; 1.47) | 0.49(-0.19; 1.17) | 0.37(-0.39; 1.13) | 0.15(-0.82; 1.12) | -0.25(-1.39; 0.90) | -0.86(-2.22; 0.51) | -1.73(-3.61; 0.15) | -2.92(-5.89; 0.04) |
| Continued smoking during pregnancy | -0.88(-1.56; -0.19) | -0.71(-1.24; -0.18) | -0.57(-1.20; 0.06) | -0.48(-1.28; 0.31) | -0.47(-1.39; 0.45) | -0.55(-1.61; 0.50) | -0.76(-2.17; 0.65) | -1.10(-3.31; 1.11) |
| BiB |  |  |  |  |  |  |  |  |
| Non-smoking | 0.41(-0.61; 1.43) | 0.36(-0.29; 1.02) | 0.32(-0.20; 0.84) | 0.28(-0.35; 0.92) | 0.25(-0.53; 1.02) | 0.22(-0.61; 1.05) | 0.21(-0.61; 1.02) | 0.20(-0.76; 1.17) |
| Quit smoking in early pregnancy | 0.34(-1.42; 2.10) | 0.33(-0.75; 1.41) | 0.30(-0.78; 1.37) | 0.23(-1.28; 1.74) | 0.11(-1.76; 1.99) | -0.07(-2.07; 1.94) | -0.33(-2.28; 1.62) | -0.69(-2.81; 1.43) |
| Continued smoking during pregnancy | -0.59(-1.98; 0.80) | -0.26(-1.16; 0.63) | 0.01(-0.69; 0.71) | 0.18(-0.66; 1.02) | 0.20(-0.82; 1.21) | 0.02(-1.05; 1.10) | -0.39(-1.45; 0.67) | -1.09(-2.39; 0.21) |
| **FL (mm)** | **12 wks** | **16 wks** | **20 wks** | **24 wks** | **28 wks** | **32 wks** | **36 wks** | **40 wks** |
| Rs1051730, per risk allele increase |  |  |  |  |  |  |  |  |
| All |  |  |  |  |  |  |  |  |
| Non-smoking | 0.02(-0.10; 0.14) | 0.02(-0.06; 0.09) | 0.02(-0.06; 0.10) | 0.04(-0.05; 0.13) | 0.06(-0.03; 0.16) | 0.10(-0.02; 0.21) | 0.14(-0.03; 0.31) | 0.19(-0.08; 0.46) |
| Quit smoking in early pregnancy | 0.03(-0.28; 0.34) | 0.01(-0.18; 0.20) | 0.00(-0.19; 0.19) | 0.00(-0.23; 0.22) | 0.00(-0.24; 0.24) | 0.00(-0.28; 0.29) | 0.02(-0.40; 0.44) | 0.04(-0.61; 0.69) |
| Continued smoking during pregnancy | 0.02(-0.24; 0.29) | -0.06(-0.21; 0.08) | -0.14(-0.27; -0.01) | -0.20(-0.35; -0.04) | -0.25(-0.41; -0.08) | -0.28(-0.48; -0.08) | -0.30(-0.59; -0.01) | -0.31(-0.77; 0.16) |
| GenR |  |  |  |  |  |  |  |  |
| Non-smoking | -0.01(-0.15; 0.12) | 0.03(-0.06; 0.12) | 0.06(-0.04; 0.17) | 0.08(-0.03; 0.19) | 0.08(-0.04; 0.20) | 0.06(-0.08; 0.21) | 0.03(-0.21; 0.27) | -0.01(-0.40; 0.38) |
| Quit smoking in early pregnancy | 0.26(-0.06; 0.59) | 0.06(-0.17; 0.29) | -0.05(-0.30; 0.21) | -0.06(-0.34; 0.23) | 0.04(-0.26; 0.34) | 0.23(-0.15; 0.61) | 0.52(-0.06; 1.11) | 0.92(-0.01; 1.84) |
| Continued smoking during pregnancy | 0.04(-0.27; 0.35) | -0.06(-0.25; 0.13) | -0.15(-0.37; 0.06) | -0.24(-0.47; 0.00) | -0.31(-0.54; -0.07) | -0.37(-0.67; -0.06) | -0.41(-0.94; 0.11) | -0.45(-1.32; 0.42) |
| BiB |  |  |  |  |  |  |  |  |
| Non-smoking | 0.22(-0.17; 0.61) | 0.07(-0.12; 0.27) | -0.01(-0.15; 0.12) | -0.05(-0.21; 0.12) | -0.02(-0.20; 0.17) | 0.07(-0.12; 0.26) | 0.22(-0.02; 0.46) | 0.42(0.02; 0.82) |
| Quit smoking in early pregnancy | -0.82(-1.47; -0.17) | -0.35(-0.70; -0.01) | -0.04(-0.33; 0.25) | 0.13(-0.23; 0.49) | 0.15(-0.26; 0.55) | 0.01(-0.42; 0.45) | -0.27(-0.84; 0.30) | -0.70(-1.59; 0.20) |
| Continued smoking during pregnancy | -0.13(-0.71; 0.45) | -0.12(-0.41; 0.17) | -0.12(-0.29; 0.05) | -0.13(-0.35; 0.09) | -0.16(-0.41; 0.10) | -0.19(-0.47; 0.08) | -0.24(-0.60; 0.12) | -0.30(-0.89; 0.30) |

**S12 Table. *Continued.***

|  | **Predicted mean difference (95% CI)** | | | | | | |
| --- | --- | --- | --- | --- | --- | --- | --- |
| **AC (mm)** | **16 wks** | **20 wks** | **24 wks** | **28 wks** | **32 wks** | **36 wks** | **40 wks** |
| Rs1051730, per risk allele increase |  |  |  |  |  |  |  |
| All |  |  |  |  |  |  |  |
| Non-smoking | -0.54(-1.07; -0.02) | -0.27(-0.64; 0.09) | 0.11(-0.36; 0.57) | 0.48(-0.14; 1.11) | 0.78(0.10; 1.46) | 0.96(0.05; 1.87) | 0.97(-0.75; 2.69) |
| Quit smoking in early pregnancy | 1.11(-0.17; 2.39) | 0.47(-0.40; 1.35) | -0.25(-1.44; 0.93) | -0.72(-2.38; 0.94) | -0.71(-2.56; 1.15) | -0.08(-2.28; 2.13) | 1.30(-2.49; 5.09) |
| Continued smoking during pregnancy | -0.22(-1.09; 0.65) | -0.59(-1.22; 0.04) | -1.03(-1.83; -0.22) | -1.33(-2.44; -0.23) | -1.39(-2.62; -0.16) | -1.11(-2.53; 0.31) | -0.43(-2.80; 1.94) |
| GenR |  |  |  |  |  |  |  |
| Non-smoking | -1.27(-2.56; 0.01) | -0.25(-0.72; 0.23) | 0.76(-0.15; 1.66) | 1.09(0.14; 2.03) | 0.36(-0.75; 1.47) | -1.71(-5.73; 2.32) | -5.32(-14.4; 3.76) |
| Quit smoking in early pregnancy | 1.01(-2.65; 4.67) | 0.79(-0.47; 2.05) | 0.46(-1.93; 2.84) | 0.09(-2.34; 2.52) | -0.26(-3.42; 2.90) | -0.55(-12.2; 11.1) | -0.77(-26.8; 25.2) |
| Continued smoking during pregnancy | -0.21(-2.55; 2.13) | -0.97(-1.95; 0.02) | -1.68(-3.42; 0.06) | -1.86(-3.70; -0.01) | -1.21(-3.32; 0.89) | 0.46(-6.71; 7.64) | 3.34(-12.8 - 19.5) |
| BiB |  |  |  |  |  |  |  |
| Non-smoking | -0.59(-1.47; 0.29) | -0.45(-1.02; 0.12) | -0.22(-1.12; 0.69) | 0.08(-1.28; 1.44) | 0.41(-1.10; 1.92) | 0.76(-0.61; 2.14) | 1.12(-0.63; 2.87) |
| Quit smoking in early pregnancy | 1.14(-0.97; 3.24) | 0.26(-0.98; 1.50) | -0.76(-2.98; 1.46) | -1.45(-4.94; 2.05) | -1.51(-5.47; 2.45) | -0.75(-4.31 - 2.80) | 0.98(-2.85; 4.82) |
| Continued smoking during pregnancy | 0.06(-1.12; 1.23) | -0.32(-1.14; 0.49) | -0.78(-1.95; 0.39) | -1.11(-2.82; 0.61) | -1.19(-3.11; 0.73) | -0.93(-2.79 - 0.93) | -0.28(-2.69; 2.13) |
| **EFW (g)** | **16 wks** | **20 wks** | **24 wks** | **28 wks** | **32 wks** | **36 wks** | **40 wks** |
| Rs1051730, per risk allele increase |  |  |  |  |  |  |  |
| All |  |  |  |  |  |  |  |
| Non-smoking | -1.8(-4.4; 0.8) | -0.5(-2.4; 1.4) | 2.4(-1.3; 6.1) | 6.6(-0.2; 13.5) | 12.1(2.1; 22.1) | 18.5(4.7; 32.3) | 25.6(4.6; 46.7) |
| Quit smoking in early pregnancy | 4.3(-1.9; 10.6) | 2.3(-2.2; 6.7) | -1.7(-11.3; 8.0) | -6.6(-24.7; 11.5) | -11.5(-37.7; 14.8) | -15.3(-51.0; 20.5) | -17.1(-70.8; 36.6) |
| Continued smoking during pregnancy | -0.6(-5.2; 4.0) | -3.7(-7.0; -0.4) | -10.0(-16.6; -3.3) | -18.4(-30.6; -6.2) | -27.9(-45.1; -10.6) | -37.2(-60.3; -14.1) | -45.4(-80.7; -10.2) |
| GenR |  |  |  |  |  |  |  |
| Non-smoking | -1.6(-4.6; 1.5) | 0.1(-2.2; 2.3) | 3.6(-0.6; 7.8) | 8.4(0.6; 16.3) | 14.3(2.5; 26.0) | 20.6(3.7; 37.5) | 26.9(0.7; 53.1) |
| Quit smoking in early pregnancy | 1.9(-5.4; 9.2) | 2.1(-3.4; 7.7) | 2.2(-8.7; 13.1) | 1.3(-19.2; 21.7) | -1.2(-32.1; 29.8) | -5.9(-51.1; 39.4) | -13.5(-84.3; 57.3) |
| Continued smoking during pregnancy | 1.5(-4.6; 7.5) | -3.6(-8.1; 0.9) | -13.1(-21.8; -4.4) | -23.8(-39.9; -7.7) | -32.9(-56.6; -9.3) | -37.4(-70.7; -4.0) | -34.2(-85.6; 17.2) |
| BiB |  |  |  |  |  |  |  |
| Non-smoking | -1.3(-6.3; 3.7) | -1.5(-4.9; 1.9) | -1.2(-9.3; 6.9) | 0.6(-14.2; 15.4) | 5.0(-14.8; 24.8) | 12.9(-11.3; 37.1) | 25.4(-10.1; 60.8) |
| Quit smoking in early pregnancy | 9.5(-1.3; 20.4) | 1.5(-5.4; 8.5) | -12.5(-30.9; 5.8) | -27.0(-61.3; 7.2) | -36.4(-83.0; 10.2) | -35.0(-92.0; 22.0) | -17.2(-98.1; 63.6) |
| Continued smoking during pregnancy | -3.0(-9.7; 3.6) | -3.3(-8.0; 1.4) | -5.2(-15.6; 5.3) | -10.0(-28.9; 8.9) | -19.1(-44.6; 6.4) | -34.0(-65.9; -2.1) | -56.0(-104.4; -7.7) |

Predicted differences in mean head circumference (HC), femur length (FL), abdominal circumference (AC) and estimated fetal weight (EFW) per maternal rs1051730 T allele increase in non-smokers, pre-pregnancy smokers quitting smoking in early pregnancy and pre-pregnancy smokers continuing smoking during pregnancy at 4-weekly gestational age intervals from 12/16 weeks through 40 weeks All mean differences (with 95% confidence intervals) are estimated using multilevel fractional polynomial models with adjustment for cohort.
