# Supplementary material for: Associations of maternal quitting, reducing, and continuing smoking during pregnancy with longitudinal fetal growth: Findings from Mendelian randomization and parental negative control studies
Source: PLoS Med. 2019 Nov 13;16(11):e1002972. doi: 10.1371/journal.pmed.1002972 (PMC6853297; doi:10.1371/journal.pmed.1002972)
Supplement: S13 Table — (DOCX) [file pmed.1002972.s025.docx]

**S13 Table. Predicted differences in mean fetal size across gestation as a proportion of the mean per risk allele increase at rs1051730 in non-smokers, pre-pregnancy smokers who quit in early pregnancy and pre-pregnancy smokers continuing smoking through pregnancy, overall and stratified by cohort.**

|  | **Predicted mean difference as a proportion of the mean** | | | | | | | |
| --- | --- | --- | --- | --- | --- | --- | --- | --- |
| **HC (mm)** | **12 wks** | **16 wks** | **20 wks** | **24 wks** | **28 wks** | **32 wks** | **36 wks** | **40 wks** |
| Rs1051730, per risk allele increase |  |  |  |  |  |  |  |  |
| All |  |  |  |  |  |  |  |  |
| Non-smoking | -0.0025 | -0.0009 | -0.0002 | 0.0002 | 0.0005 | 0.0007 | 0.0009 | 0.0011 |
| Quit smoking in early pregnancy | 0.0061 | 0.0032 | 0.0019 | 0.0009 | -0.0001 | -0.0012 | -0.0026 | -0.0043 |
| Continued smoking during pregnancy | -0.0092 | -0.0034 | -0.0013 | -0.0005 | -0.0004 | -0.0008 | -0.0017 | -0.0031 |
| GenR |  |  |  |  |  |  |  |  |
| Non-smoking | -0.0031 | -0.0019 | -0.0013 | -0.0008 | -0.0002 | 0.0004 | 0.0012 | 0.0023 |
| Quit smoking in early pregnancy | 0.0074 | 0.0038 | 0.0021 | 0.0007 | -0.0009 | -0.0028 | -0.0053 | -0.0085 |
| Continued smoking during pregnancy | -0.0130 | -0.0059 | -0.0033 | -0.0022 | -0.0017 | -0.0018 | -0.0023 | -0.0032 |
| BiB |  |  |  |  |  |  |  |  |
| Non-smoking | 0.0063 | 0.0031 | 0.0019 | 0.0012 | 0.0009 | 0.0007 | 0.0009 | 0.0006 |
| Quit smoking in early pregnancy | 0.0048 | 0.0027 | 0.0018 | 0.0012 | 0.0006 | 0.0000 | -0.0026 | -0.0016 |
| Continued smoking during pregnancy | -0.0098 | -0.0026 | 0.0000 | 0.0009 | 0.0009 | 0.0003 | -0.0017 | -0.0032 |
| **FL (mm)** | **12 wks** | **16 wks** | **20 wks** | **24 wks** | **28 wks** | **32 wks** | **36 wks** | **40 wks** |
| Rs1051730, per risk allele increase |  |  |  |  |  |  |  |  |
| All |  |  |  |  |  |  |  |  |
| Non-smoking | 0.0034 | 0.0008 | 0.0007 | 0.0009 | 0.0012 | 0.0016 | 0.0021 | 0.0027 |
| Quit smoking in early pregnancy | 0.0050 | 0.0007 | 0.0000 | -0.0001 | 0.0000 | 0.0002 | 0.0005 | 0.0008 |
| Continued smoking during pregnancy | 0.0035 | -0.0032 | -0.0043 | -0.0046 | -0.0047 | -0.0046 | -0.0045 | -0.0042 |
| GenR |  |  |  |  |  |  |  |  |
| Non-smoking | -0.0022 | 0.0016 | 0.0019 | 0.0018 | 0.0015 | 0.0011 | 0.0006 | 0.0000 |
| Quit smoking in early pregnancy | 0.0466 | 0.0027 | -0.0016 | -0.0013 | 0.0008 | 0.0040 | 0.0080 | 0.0128 |
| Continued smoking during pregnancy | 0.0072 | -0.0037 | -0.0052 | -0.0058 | -0.006 | -0.0061 | -0.0061 | -0.0061 |
| BiB |  |  |  |  |  |  |  |  |
| Non-smoking | 0.0348 | 0.0039 | -0.0005 | -0.0011 | -0.0003 | 0.0012 | 0.0021 | 0.0058 |
| Quit smoking in early pregnancy | -0.1287 | -0.0178 | -0.0007 | 0.0036 | 0.0033 | 0.0006 | 0.0005 | -0.0095 |
| Continued smoking during pregnancy | -0.0241 | -0.0065 | -0.0037 | -0.0029 | -0.0028 | -0.0031 | -0.0045 | -0.0043 |

**S13 Table. *Continued.***

|  | **Predicted mean difference as a proportion of the mean** | | | | | | |
| --- | --- | --- | --- | --- | --- | --- | --- |
| **AC (mm)** | **16 wks** | **20 wks** | **24 wks** | **28 wks** | **32 wks** | **36 wks** | **40 wks** |
| Rs1051730, per risk allele increase |  |  |  |  |  |  |  |
| All |  |  |  |  |  |  |  |
| Non-smoking | -0.0046 | -0.0018 | 0.0006 | 0.0021 | 0.0029 | 0.0031 | 0.0030 |
| Quit smoking in early pregnancy | 0.0093 | 0.0034 | -0.0009 | -0.0026 | -0.0022 | 0.0000 | 0.0040 |
| Continued smoking during pregnancy | -0.0024 | -0.0038 | -0.0047 | -0.0048 | -0.0043 | -0.0031 | -0.0013 |
| GenR |  |  |  |  |  |  |  |
| Non-smoking | -0.0103 | -0.0016 | 0.0038 | 0.0045 | 0.0014 | -0.0050 | -0.0156 |
| Quit smoking in early pregnancy | 0.0082 | 0.0052 | 0.0025 | 0.0006 | -0.0007 | -0.0017 | -0.0026 |
| Continued smoking during pregnancy | -0.0016 | -0.0060 | -0.0080 | -0.0070 | -0.0036 | 0.0022 | 0.0111 |
| BiB |  |  |  |  |  |  |  |
| Non-smoking | -0.0052 | -0.0031 | -0.0010 | 0.0005 | 0.0016 | 0.0031 | 0.0034 |
| Quit smoking in early pregnancy | 0.0089 | 0.0025 | -0.0022 | -0.0041 | -0.0036 | 0.0000 | 0.0031 |
| Continued smoking during pregnancy | -0.0005 | -0.0022 | -0.0035 | -0.0038 | -0.0035 | -0.0031 | -0.0008 |
| **EFW (g)** | **16 wks** | **20 wks** | **24 wks** | **28 wks** | **32 wks** | **36 wks** | **40 wks** |
| Rs1051730, per risk allele increase |  |  |  |  |  |  |  |
| All |  |  |  |  |  |  |  |
| Non-smoking | -0.0101 | -0.0016 | 0.0031 | 0.0051 | 0.0061 | 0.0067 | 0.0072 |
| Quit smoking in early pregnancy | 0.0206 | 0.0071 | -0.0002 | -0.0031 | -0.0044 | -0.0051 | -0.0055 |
| Continued smoking during pregnancy | -0.0043 | -0.0107 | -0.0135 | -0.0142 | -0.0141 | -0.0135 | -0.0126 |
| GenR |  |  |  |  |  |  |  |
| Non-smoking | -0.0107 | -0.0002 | 0.005 | 0.0069 | 0.0076 | 0.0078 | 0.0078 |
| Quit smoking in early pregnancy | 0.0121 | 0.0071 | 0.0036 | 0.0013 | -0.0007 | -0.0027 | -0.0048 |
| Continued smoking during pregnancy | 0.0064 | -0.0113 | -0.0184 | -0.019 | -0.0169 | -0.0136 | -0.0093 |
| BiB |  |  |  |  |  |  |  |
| Non-smoking | -0.0089 | -0.0045 | -0.0012 | 0.001 | 0.0028 | 0.0047 | 0.0066 |
| Quit smoking in early pregnancy | 0.0434 | 0.0072 | -0.01 | -0.0141 | -0.0134 | -0.0103 | -0.0057 |
| Continued smoking during pregnancy | -0.0176 | -0.0107 | -0.0084 | -0.0088 | -0.0103 | -0.0126 | -0.0154 |

Predicted differences in mean head circumference (HC), femur length (FL), abdominal circumference (AC) and estimated fetal weight (EFW) as a proportion of the mean per maternal rs1051730 T allele increase by maternal smoking status and at 4-weekly gestational age intervals from 12/16 weeks through 40 weeks. All mean differences (with 95% confidence intervals) are estimated using multilevel fractional polynomial models with adjustment for cohort.
