# Supplementary material for: Associations of maternal quitting, reducing, and continuing smoking during pregnancy with longitudinal fetal growth: Findings from Mendelian randomization and parental negative control studies
Source: PLoS Med. 2019 Nov 13;16(11):e1002972. doi: 10.1371/journal.pmed.1002972 (PMC6853297; doi:10.1371/journal.pmed.1002972)
Supplement: S14 Table — (DOCX) [file pmed.1002972.s026.docx]

**S14 Table. Predicted differences in mean fetal size (with 95% CIs) across gestation per risk allele increase at rs1051730 in pre-pregnancy smokers and non-smokers, overall and stratified by cohort.**

|  | **Predicted mean difference (95% CI)** | | | | | | | |
| --- | --- | --- | --- | --- | --- | --- | --- | --- |
| **HC (mm)** | **12 wks** | **16 wks** | **20 wks** | **24 wks** | **28 wks** | **32 wks** | **36 wks** | **40 wks** |
| Rs1051730, per risk allele increase |  |  |  |  |  |  |  |  |
| All |  |  |  |  |  |  |  |  |
| Non-smoking | -0.19 (-0.50;0.13) | -0.11 (-0.35;0.13) | -0.04 (-0.30;0.23) | 0.04 (-0.30;0.38) | 0.12 (-0.27;0.51) | 0.20 (-0.22;0.63) | 0.29 (-0.21;0.79) | 0.38 (-0.34; 1.10) |
| Smoking | -0.24 (-0.75;0.26) | -0.14 (-0.50;0.23) | -0.07 (-0.45;0.32) | -0.06 (-0.55;0.42) | -0.16 (-0.73;0.41) | -0.38 (-1.00;0.23) | -0.77 (-1.46;-0.08) | -1.34 (-2.29;-0.39) |
| GenR |  |  |  |  |  |  |  |  |
| Non-smoking | -0.21 (-0.54;0.12) | -0.23 (-0.48;0.02) | -0.22 (-0.52;0.08) | -0.17 (-0.56;0.22) | -0.07 (-0.52;0.38) | 0.11 (-0.40;0.62) | 0.38 (-0.30;1.06) | 0.75 (-0.32 ;1.82) |
| Smoking | -0.33 (-0.88;0.23) | -0.27 (-0.69;0.15) | -0.26 (-0.75;0.23) | -0.31 (-0.93;0.31) | -0.47 (-1.20;0.25) | -0.76 (-1.60;0.08) | -1.22 (-2.35;-0.08) | -1.87 (-3.65;-0.08) |
| BiB |  |  |  |  |  |  |  |  |
| Non-smoking | 0.41 (-0.61;1.43) | 0.36 (-0.29;1.02) | 0.32 (-0.20;0.84) | 0.28 (-0.35 ;0.92) | 0.25 (-0.53;1.02) | 0.22 (-0.61;1.05) | 0.21 (-0.61;1.02) | 0.20 (-0.76;1.17) |
| Smoking | -0.34 (-1.47;0.79) | -0.10 (-0.82;0.62) | 0.09 (-0.50;0.68) | 0.20 (-0.54;0.93) | 0.18 (-0.72;1.08) | 0.00 (-0.96;0.96) | -0.38 (-1.32;0.56) | -0.99 (-2.10;0.13) |
| **FL (mm)** | **12 wks** | **16 wks** | **20 wks** | **24 wks** | **28 wks** | **32 wks** | **36 wks** | **40 wks** |
| Rs1051730, per risk allele increase |  |  |  |  |  |  |  |  |
| All |  |  |  |  |  |  |  |  |
| Non-smoking | 0.02 (-0.10;0.14) | 0.02 (-0.06;0.09) | 0.02 (-0.06;0.10) | 0.04 (-0.05;0.13) | 0.06 (-0.03;0.16) | 0.10 (-0.02;0.21) | 0.14 (-0.03;0.31) | 0.19 (-0.08;0.46) |
| Smoking | 0.03 (-0.17;0.23) | -0.04 (-0.16;0.08) | -0.10 (-0.21;0.01) | -0.15 (-0.28;-0.02) | -0.18 (-0.32;-0.05) | -0.21 (-0.37;-0.04) | -0.22 (-0.45;0.02) | -0.21 (-0.59;0.17) |
| GenR |  |  |  |  |  |  |  |  |
| Non-smoking | -0.01 (-0.15;0.12) | 0.03 (-0.06;0.12) | 0.06 (-0.04;0.17) | 0.08 (-0.03;0.19) | 0.08 (-0.04;0.20) | 0.06 (-0.08;0.21) | 0.03 (-0.21;0.27) | -0.01 (-0.40;0.38) |
| Smoking | 0.15 (-0.07;0.38) | -0.01 (-0.15;0.14) | -0.12 (-0.29;0.04) | -0.19 (-0.37;0.00) | -0.21 (-0.39;-0.02) | -0.18 (-0.42;0.06) | -0.10 (-0.50;0.29) | 0.02 (-0.63;0.66) |
| BiB |  |  |  |  |  |  |  |  |
| Non-smoking | 0.22 (-0.17;0.61) | 0.07 (-0.12;0.27) | -0.01 (-0.15;0.12) | -0.05 (-0.21;0.12) | -0.02 (-0.20;0.17) | 0.07 (-0.12;0.26) | 0.22 (-0.02;0.46) | 0.42 (0.02;0.82) |
| Smoking | -0.30 (-0.75;0.15) | -0.18 (-0.40;0.04) | -0.11(-0.26;0.04) | -0.07 (-0.26;0.11) | -0.09 (-0.30;0.13) | -0.14 (-0.37;0.09) | -0.24 (-0.55;0.06) | -0.38 (-0.87; 0.11) |

**S14 Table. *Continued.***

|  | **Predicted mean difference (95% CI)** | | | | | | |
| --- | --- | --- | --- | --- | --- | --- | --- |
| **AC (mm)** | **16 wks** | **20 wks** | **24 wks** | **28 wks** | **32 wks** | **36 wks** | **40 wks** |
| Rs1051730, per risk allele increase |  |  |  |  |  |  |  |
| All |  |  |  |  |  |  |  |
| Non-smoking | -0.54 (-1.07;-0.02) | -0.27 (-0.64;0.09) | 0.11(-0.36;0.57) | 0.48 (-0.14;1.11) | 0.78 (0.10;1.46) | 0.96 (0.05;1.87) | 0.97 (-0.75;2.69) |
| Smoking | 0.16 (-0.56; 0.88) | -0.27 (-0.78;0.24) | -0.78 (-1.45;-0.12) | -1.15 (-2.07;-0.23) | -1.23 (-2.26;-0.21) | -0.94 (-2.15;0.27) | -0.18 (-2.22;1.85) |
| GenR |  |  |  |  |  |  |  |
| Non-smoking | -1.27 (-2.56;0.01) | -0.25 (-0.72;0.23) | 0.76 (-0.15;1.66) | 1.09 (0.14;2.03) | 0.36 (-0.75;1.47) | -1.71 (-5.73;2.32) | -5.32 (-14.4;3.76) |
| Smoking | -0.26 (-2.31;1.79) | -0.41 (-1.19;0.36) | -0.68 (-2.14;0.78) | -1.02 (-2.52;0.49) | -1.40 (-3.24; 0.44) | -1.81 (-8.36;4.73) | -2.24 (-16.9;12.4) |
| BiB |  |  |  |  |  |  |  |
| Non-smoking | -0.59 (-1.47;0.29) | -0.45 (-1.02;0.12) | -0.22 (-1.12;0.69) | 0.08 (-1.28;1.44) | 0.41 (-1.10;1.92) | 0.76 (-0.61;2.14) | 1.12 (-0.63;2.87) |
| Smoking | 0.22 (-0.81;1.24) | -0.16 (-0.84;0.53) | -0.60 (-1.65;0.44) | -0.91 (-2.48;0.65) | -0.98 (-2.74; 0.79) | -0.71 (-2.38;0.97) | -0.03 (-2.11;2.04) |
| **EFW (g)** | **16 wks** | **20 wks** | **24 wks** | **28 wks** | **32 wks** | **36 wks** | **40 wks** |
| Rs1051730, per risk allele increase |  |  |  |  |  |  |  |
| All |  |  |  |  |  |  |  |
| Non-smoking | -1.8 (-4.4;0.8) | -0.5 (-2.4;1.4) | 2.4 (-1.3;6.1) | 6.6 (-0.2;13.5) | 12.1 (2.1;22.1) | 18.5 (4.7;32.3) | 25.6 (4.6;46.7) |
| Smoking | 0.9 (-2.8;4.6) | -1.9 (-4.6;0.8) | -7.6 (-13.1;-2.1) | -15.2 (-25.3;-5.0) | -23.8 (-38.3;-9.3) | -32.4 (-52.0;-12.8) | -40.1 (-69.9;-10.3) |
| GenR |  |  |  |  |  |  |  |
| Non-smoking | -1.6 (-4.6;1.5) | 0.1 (-2.2;2.3) | 3.6 (-0.6;7.8) | 8.4 (0.6;16.3) | 14.3 (2.5;26.0) | 20.6 (3.7;37.5) | 26.9 (0.7;53.1) |
| Smoking | 1.7 (-3.0;6.4) | -1.6 (-5.1;1.9) | -8.1 (-15.0;-1.2) | -16.1(-28.8;-3.3) | -23.9 (-42.9;-4.9) | -29.9 (-57.1;-2.7) | -32.5 (-74.5;9.5) |
| BiB |  |  |  |  |  |  |  |
| Non-smoking | -1.3 (-6.3;3.7) | -1.5 (-4.9;1.9) | -1.2 (-9.3;6.9) | 0.6 (-14.2;15.4) | 5.0 (-14.8;24.8) | 12.9 (-11.3;37.1) | 25.4 (-10.1;60.8) |
| Smoking | -0.6 (-6.3;5.1) | -2.0 (-5.9;1.9) | -5.3 (-14.4;3.8) | -10.9 (-27.6;5.7) | -19.1 (-41.8;3.6) | -30.1 (-58.5;-1.7) | -44.2 (-86.5;-1.8) |

Predicted differences in mean head circumference (HC), femur length (FL), abdominal circumference (AC) and estimated fetal weight (EFW) per maternal rs1051730 T allele increase in pre-pregnancy smokers and non-smokers at 4-weekly gestational age intervals from 12/16 weeks through 40 weeks. All mean differences (with 95% confidence intervals) are estimated using multilevel fractional polynomial models with adjustment for cohort.
