# Supplementary material for: Associations of maternal quitting, reducing, and continuing smoking during pregnancy with longitudinal fetal growth: Findings from Mendelian randomization and parental negative control studies
Source: PLoS Med. 2019 Nov 13;16(11):e1002972. doi: 10.1371/journal.pmed.1002972 (PMC6853297; doi:10.1371/journal.pmed.1002972)
Supplement: S15 Table — (DOCX) [file pmed.1002972.s027.docx]

**S15 Table. Association of maternal rs10151730 genotype with potential confounders.**

|  | **Non-smokers** | | **Pre-pregnancy smokers**  **quitting smoking before the second trimester** | | **Pre-pregnancy smokers continuing smoking during pregnancy** | |
| --- | --- | --- | --- | --- | --- | --- |
| **GenR** |  |  |  |  |  |  |
| Maternal characteristics | N | Beta (95% CI) | N | Beta (95% CI) | N | Beta (95% CI) |
| Age (years) | 2622 | 0.09 (-0.15; 0.33) | 332 | 0.07 (-0.62; 0.76) | 650 | 0.47 (-0.17; 1.11) |
| Height (cm) | 2622 | 0.09 (-0.28; 0.47) | 332 | -0.56 (-1.59; 0.46) | 649 | -0.43 (-1.17; 0.31) |
| Body mass index (kg/m^2^) | 2344 | -0.05 (-0.29; 0.18) | 301 | -0.05 (-0.63; 0.53) | 561 | -0.17 (-0.73; 0.38) |
| Multiparous (yes vs. no) | 2619 | 0.04 (-0.08; 0.15) | 331 | 0.02 (-0.33; 0.36) | 647 | -0.21 (-0.44; 0.02) |
| High education (yes vs. no) | 2611 | 0.07 (-0.05; 0.19) | 331 | 0.18 (-0.14; 0.50) | 632 | 0.21 (-0.06; 0.47) |
| Alcohol (yes vs. no) | 2620 | 0.02 (-0.10; 0.14) | 332 | -0.05 (-0.47; 0.37) | 609 | 0.19 (-0.06; 0.44) |
| **BiB** |  |  |  |  |  |  |
| Maternal characteristics | N | Beta (95% CI) | N | Beta (95% CI) | N | Beta (95% CI) |
| Age (years) | 1675 | -0.09 (-0.52; 0.33) | 356 | 0.39 (-0.50; 1.27) | 892 | 0.38 (-0.17; 0.93) |
| Height (cm) | 1649 | -0.22 (-0.68; 0.23) | 353 | -0.29 (-1.29; 0.70) | 880 | -0.12 (-0.74; 0.50) |
| Body mass index (kg/m^2^) | 1602 | 0.12 (-0.33; 0.57) | 348 | -0.01 (-0.93; 0.91) | 853 | -0.13 (-0.74; 0.48) |
| Multiparous (yes vs. no) | 1633 | -0.03 (-0.18; 0.11) | 351 | -0.49 (-0.84; -0.14) | 868 | 0.10 (-0.10; 0.30) |
| High education (yes vs. no) | 1497 | 0.03 (-0.12; 0.18) | 326 | 0.28 (-0.07; 0.62) | 823 | 0.07 (-0.18; 0.33) |
| Alcohol (yes vs. no) | 1670 | -0.10 (-0.25; 0.05) | 356 | 0.14 (-0.22; 0.50) | 892 | -0.01 (-0.22; 0.20) |
| **All** |  |  |  |  |  |  |
| Maternal characteristics | N | Beta (95% CI) | N | Beta (95% CI) | N | Beta (95% CI) |
| Age (years) | 4297 | 0.02 (-0.20; 0.24) | 688 | 0.23 (-0.34; 0.79) | 1542 | 0.42 (0.00; 0.84) |
| Height (cm) | 4271 | -0.03 (-0.31; 0.26) | 685 | -0.43 (-1.14; 0.28) | 1529 | -0.25 (-0.73; 0.23) |
| Body mass index (kg/m^2^) | 3946 | 0.02 (-0.21; 0.24) | 649 | -0.03 (-0.58; 0.53) | 1414 | -0.15 (-0.57; 0.28) |
| Multiparous (yes vs. no) | 4252 | 0.01 (-0.08; 0.10) | 682 | -0.24 (-0.49; 0.00) | 1515 | -0.03 (-0.18; 0.12) |
| High education (yes vs. no) | 4108 | 0.05 (-0.04; 0.15) | 657 | 0.22 (-0.01; 0.46) | 1455 | 0.14 (-0.05; 0.32) |
| Alcohol (yes vs. no) | 4290 | -0.03 (-0.12; 0.07) | 688 | 0.06 (-0.21; 0.33) | 1501 | 0.07 (-0.09; 0.23) |

Association of maternal rs1051730 genotype with potential confounders, overall and by cohort. Betas represent differences in maternal characteristics per T allele increase at rs1051730. Pooled analyses (all) were adjusted for cohort.
