# Supplementary material for: Associations of maternal quitting, reducing, and continuing smoking during pregnancy with longitudinal fetal growth: Findings from Mendelian randomization and parental negative control studies
Source: PLoS Med. 2019 Nov 13;16(11):e1002972. doi: 10.1371/journal.pmed.1002972 (PMC6853297; doi:10.1371/journal.pmed.1002972)
Supplement: S16 Table — (DOCX) [file pmed.1002972.s028.docx]

**S16 Table. Parental smoking during pregnancy and predicted differences in mean fetal size (with 95% CIs) across gestation, overall and stratified by cohort.**

|  | **Predicted mean difference (95% CI)** | | | | | | | |
| --- | --- | --- | --- | --- | --- | --- | --- | --- |
| **HC (mm)** | **12 wks** | **16 wks** | **20 wks** | **24 wks** | **28 wks** | **32 wks** | **36 wks** | **40 wks** |
|  |  |  |  |  |  |  |  |  |
| All |  |  |  |  |  |  |  |  |
| Maternal smoking during pregnancy | -0.15 (-0.71; 0.40) | -0.34 (-0.75; 0.06) | -0.60 (-1.04;-0.15) | -0.96 (-1.51;-0.40) | -1.47 (-2.11;-0.84) | -2.20 (-2.89;-1.50) | -3.17 (-4.02;-2.32) | -4.44 (-5.73;-3.15) |
| Partner smoking during pregnancy | 0.45 (0.05; 0.85) | 0.45 (0.15; 0.75) | 0.44 (0.10; 0.77) | 0.40 (-0.02; 0.82) | 0.33 (-0.16; 0.81) | 0.21 (-0.32; 0.74) | 0.03 (-0.63; 0.70) | -0.21 (-1.22; 0.80) |
| GenR |  |  |  |  |  |  |  |  |
| Maternal smoking during pregnancy | -0.13 (-0.70; 0.44) | -0.43 (-0.85;-0.01) | -0.75 (-1.24;-0.27) | -1.12 (-1.73;-0.52) | -1.56 (-2.25;-0.86) | -2.08 (-2.86;-1.29) | -2.71 (-3.77;-1.65) | -3.46 (-5.16;-1.76) |
| Partner smoking during pregnancy | 0.39 (-0.01; 0.79) | 0.39 (0.09; 0.70) | 0.37 (0.01; 0.72) | 0.30 (-0.15; 0.74) | 0.16 (-0.35; 0.67) | -0.05 (-0.63; 0.53) | -0.37 (-1.16; 0.41) | -0.82 (-2.07; 0.44) |
| BiB |  |  |  |  |  |  |  |  |
| Maternal smoking during pregnancy | -0.95 (-3.11; 1.20) | -0.55 (-1.94; 0.85) | -0.31 (-1.42; 0.79) | -0.39 (-1.71; 0.94) | -0.91 (-2.50; 0.69) | -2.01 (-3.70;-0.33) | -3.83 (-5.47;-2.20) | -6.52 (-8.46;-4.57) |
| Partner smoking during pregnancy | 1.53 (-0.27; 3.34) | 1.19 (0.02; 2.37) | 0.90 (-0.04; 1.85) | 0.70 (-0.44; 1.85) | 0.64 (-0.75; 2.03) | 0.74 (-0.73; 2.22) | 1.06 (-0.39; 2.51) | 1.63 (-0.08; 3.33) |
| **FL (mm)** | **12 wks** | **16 wks** | **20 wks** | **24 wks** | **28 wks** | **32 wks** | **36 wks** | **40 wks** |
|  |  |  |  |  |  |  |  |  |
| All |  |  |  |  |  |  |  |  |
| Maternal smoking during pregnancy | -0.23 (-0.45;-0.01) | -0.21 (-0.35;-0.08) | -0.25 (-0.39;-0.11) | -0.35 (-0.51;-0.20) | -0.51 (-0.68;-0.35) | -0.73 (-0.93;-0.54) | -1.01 (-1.32;-0.71) | -1.35 (-1.85;-0.85) |
| Partner smoking during pregnancy | 0.04 (-0.12; 0.20) | 0.11 (0.01; 0.21) | 0.15 (0.04; 0.25) | 0.16 (0.04; 0.28) | 0.14 (0.02; 0.26) | 0.10 (-0.05; 0.25) | 0.02 (-0.21; 0.26) | -0.08 (-0.46; 0.31) |
| GenR |  |  |  |  |  |  |  |  |
| Maternal smoking during pregnancy | -0.31 (-0.55;-0.07) | -0.28 (-0.43;-0.13) | -0.31 (-0.48;-0.15) | -0.41 (-0.59;-0.23) | -0.57 (-0.75;-0.39) | -0.79 (-1.02;-0.56) | -1.07 (-1.46;-0.68) | -1.42 (-2.06;-0.77) |
| Partner smoking during pregnancy | 0.02 (-0.15; 0.19) | 0.10 (-0.00; 0.21) | 0.15 (0.03; 0.27) | 0.16 (0.03; 0.30) | 0.14 (0.00; 0.27) | 0.08 (-0.09; 0.25) | -0.02 (-0.30; 0.27) | -0.15 (-0.61; 0.32) |
| BiB |  |  |  |  |  |  |  |  |
| Maternal smoking during pregnancy | 0.23 (-0.58; 1.04) | 0.12 (-0.29; 0.52) | -0.02 (-0.30; 0.26) | -0.18 (-0.53; 0.17) | -0.37 (-0.76; 0.02) | -0.58 (-0.97;-0.19) | -0.82 (-1.33;-0.30) | -1.08 (-1.95;-0.21) |
| Partner smoking during pregnancy | -0.03 (-0.69; 0.62) | 0.07 (-0.27; 0.40) | 0.13 (-0.11; 0.37) | 0.16 (-0.14; 0.45) | 0.15 (-0.18; 0.48) | 0.11 (-0.24; 0.45) | 0.03 (-0.43; 0.48) | -0.09 (-0.84; 0.66) |

**S16 Table. *Continued.***

|  | **Predicted mean difference (95% CI)** | | | | | | |
| --- | --- | --- | --- | --- | --- | --- | --- |
| **AC (mm)** | **16 wks** | **20 wks** | **24 wks** | **28 wks** | **32 wks** | **36 wks** | **40 wks** |
|  |  |  |  |  |  |  |  |
| All |  |  |  |  |  |  |  |
| Maternal smoking during pregnancy | 0.33 (-0.35; 1.01) | -0.08 (-0.55; 0.40) | -0.84 (-1.44;-0.23) | -1.86 (-2.69; -1.03) | -3.09 (-4.00;-2.19) | -4.50 (-5.55 -3.45) | -6.05 (-7.87; -4.23) |
| Partner smoking during pregnancy | 0.04 (-0.70; 0.77) | 0.40 (-0.09; 0.89) | 0.77 (0.16; 1.39) | 0.94 (0.15; 1.73) | 0.76 (-0.10; 1.63) | 0.16 (-1.30; 1.61) | -0.96 (-3.95; 2.03) |
| GenR |  |  |  |  |  |  |  |
| Maternal smoking during pregnancy | -0.04 (-2.08; 2.01) | -0.31 (-1.03; 0.41) | -0.89 (-2.30; 0.52) | -1.75 (-3.19; -0.31) | -2.87 (-4.58;-1.16) | -4.24 (-10.66; 2.17) | -5.86 (-20.35; 8.64) |
| Partner smoking during pregnancy | 0.09 (-1.52; 1.70) | 0.40 (-0.17; 0.97) | 0.73 (-0.37; 1.83) | 0.90 (-0.25; 2.04) | 0.80 (-0.52; 2.12) | 0.37 (-4.58; 5.32) | -0.47 (-11.69; 10.74) |
| BiB |  |  |  |  |  |  |  |
| Maternal smoking during pregnancy | 0.77 (-0.22; 1.75) | 0.14 (-0.50; 0.79) | -0.88 (-1.85; 0.09) | -2.10 (-3.55; -0.66) | -3.41 (-5.02;-1.81) | -4.72 (-6.19; -3.25) | -5.96 (-7.80; -4.12) |
| Partner smoking during pregnancy | -0.00 (-1.58; 1.57) | 0.40 (-0.67; 1.46) | 0.82 (-0.79; 2.43) | 1.03 (-1.35; 3.41) | 0.88 (-1.77; 3.53) | 0.27 (-2.21; 2.75) | -0.88 (-4.01; 2.25) |
| **EFW (g)** | **16 wks** | **20 wks** | **24 wks** | **28 wks** | **32 wks** | **36 wks** | **40 wks** |
|  |  |  |  |  |  |  |  |
| All |  |  |  |  |  |  |  |
| Maternal smoking during pregnancy | -1.4 (-4.7; 1.9) | -2.6 (-5.1; -0.1) | -9.8 (-15.1; -4.4) | -28.4 (-38.0; -18.7) | -63.8 (-77.1;-50.4) | -121.4 (-138.3;-104.4) | -206.6 (-231.3;-181.9) |
| Partner smoking during pregnancy | 0.6 (-2.5; 3.7) | 2.8 (0.4 - 5.2) | 6.5 (1.6; 11.4) | 9.9 (0.9; 18.8) | 11.2 (-1.7; 24.1) | 8.7 (-9.0; 26.3) | 0.5 (-25.9: 26.9) |
| GenR |  |  |  |  |  |  |  |
| Maternal smoking during pregnancy | -0.2 (-4.4; 4.0) | -3.2 (-6.5; 0.1) | -12.6 (-19.1; -6.0) | -31.0 (-43.0; -19.1) | -61.5 (-78.7;-44.2) | -106.7 (-130.3; -83.0) | -169.4 (-204.7; -134.1) |
| Partner smoking during pregnancy | 0.8 (-2.5; 4.1) | 2.7 (0.1; 5.3) | 5.8 (0.6; 11.0) | 8.8 (-0.8; 18.3) | 10.1 (-3.9; 24.0) | 8.3 (-11.1; 27.7) | 2.0 (-27.2; 31.2) |
| BiB |  |  |  |  |  |  |  |
| Maternal smoking during pregnancy | -4.9 (-10.5; 0.6) | -1.2 (-5.0; 2.6) | -0.9 (-10.7; 8.8) | -14.6 (-32.1; 3.0) | -52.6 (-75.3;-29.8) | -125.3 (-150.8; -99.6) | -242.9 (-278.0; -207.8) |
| Partner smoking during pregnancy | 0.8 (-7.7; 9.3) | 4.2 (-2.1; 10.4) | 9.9 (-6.1; 26.0) | 15.5 (-13.2; 44.2) | 18.3 (-19.3; 55.8) | 15.7 (-28.3; 59.6) | 5.1 (-57.8; 68.0) |

Predicted differences in mean head circumference (HC), femur length (FL), abdominal circumference (AC) and estimated fetal weight (EFW) associated with maternal smoking (i.e. comparing maternal continued smoking through pregnancy with no maternal smoking during pregnancy (= reference category)) and mother’s partner’s smoking (comparing partner smoking during pregnancy with no partner smoking during pregnancy (= reference category)) at 4-weekly gestational age intervals from 12/16 weeks through 40 weeks. All mean differences (with 95% confidence intervals) are estimated using multilevel fractional polynomial models with adjustment for cohort, infant sex, maternal parity, maternal and partner’s age, height, body mass index, education, alcohol use during pregnancy and maternal smoking during pregnancy.
