# Supplementary material for: Associations of maternal quitting, reducing, and continuing smoking during pregnancy with longitudinal fetal growth: Findings from Mendelian randomization and parental negative control studies
Source: PLoS Med. 2019 Nov 13;16(11):e1002972. doi: 10.1371/journal.pmed.1002972 (PMC6853297; doi:10.1371/journal.pmed.1002972)
Supplement: S18 Table — (DOCX) [file pmed.1002972.s030.docx]

**S18 Table. Predicted differences in mean infant birth weight in grams (with 95% CIs) associated with maternal smoking during pregnancy and smoking intensity in continued smokers.**

|  | **Mean difference (95% CI) in grams** | |
| --- | --- | --- |
| **Maternal smoking status** | **Model 1** | **Model 2** |
| Non-smokers | REF | REF |
| Pre-pregnancy smokers who quit smoking in early pregnancy | 13.4 (-25.7; 52.4) | 3.4 (-27.9; 34.7) |
| Pre-pregnancy smokers who continue smoking during pregnancy | -247.5 (-277.6;-217.4) | -213.2 (-237.4;-189.1) |
| **Maternal smoking intensity in continued smokers** |  |  |
| Non-smoking | REF | REF |
| Low | -156.6 (-203.6;-109.7) | -151.2 (-188.7;-113.7) |
| Moderate | -253.6 (-293.6;-213.7) | -220.3 (-252.2;-188.4) |
| Heavy | -365.4 (-417.8;-312.9) | -290.3 (-332.2;-248.3) |
| **Partner smoking status *** |  |  |
| Non-smokers | REF | REF |
| Smokers | -9.6 (-41.1; 21.8) | -0.1 (-25.6; 25.5) |
| **Partner smoking intensity *** |  |  |
| Non-smoking | REF | REF |
| Low | -17.0 (-60.7; 26.6) | -13.1 (-48.6; 22.4) |
| Moderate | 14.4 (-39.1; 67.8) | 21.4 (-22.1; 64.9) |
| Heavy | -12.2 (-53.6; 29.2) | 4.9 (-28.7; 38.6) |
| **Maternal rs1051730 (per risk allele increase) **** |  |  |
| Non-smokers | 23.9 (-0.4; 48.2) | 13.4 (-13.7; 40.5) |
| Pre-pregnancy smokers who quit smoking in early pregnancy | -13.3 (-72.4; 45.7) | 4.8 (-65.7; 75.4) |
| Pre-pregnancy smokers who continue smoking during pregnancy | -52.7 (-93.0; 12.5) | -40.5 (-87.3; 6.3) |

model 1: adjusting for cohort, maternal age, parity, height, body mass index, education level and alcohol use

model 2: adjusting for cohort, maternal age, parity, height, body mass index, education level and alcohol use and gestational age at birth.

*models 1 and 2 additionally adjusted for maternal smoking during pregnancy. **models 1 excluding age, parity, height, body mass index, education level and alcohol use.
