# Supplementary material for: Associations of maternal quitting, reducing, and continuing smoking during pregnancy with longitudinal fetal growth: Findings from Mendelian randomization and parental negative control studies
Source: PLoS Med. 2019 Nov 13;16(11):e1002972. doi: 10.1371/journal.pmed.1002972 (PMC6853297; doi:10.1371/journal.pmed.1002972)
Supplement: S1 Text — (DOCX) [file pmed.1002972.s031.docx]

**S1 Text. Supplemental Methods.**

**Study cohorts**

***Generation R (GenR)***

The Generation R study (GenR) is a population-based prospective cohort study of pregnant women and their offspring in a multi-ethnic urban population in Rotterdam, the Netherlands that enrolled 9778 women (response rate = 61%) [1]. The cohort is largely representative of the female population in Rotterdam Partners from mothers enrolled in pregnancy were also invited to participate. The study has collected detailed information on both offspring and their parents through questionnaires, biological sampling and physical examinations. Fetal ultrasound examinations were carried out at research centers in early, mid- and late pregnancy and information on birth anthropometrics (weight and head circumference) was obtained through medical records. Head circumference was not routinely measured at birth, as such neonatal HC was not available for all offspring.

Information on maternal smoking before and during pregnancy was obtained by self-completed questionnaires. At enrolment, each mother was asked whether she smoked during pregnancy (no smoking, smoking until pregnancy was acknowledged (early pregnancy only smoking), continued smoking during pregnancy). This questionnaire was sent to all mothers independent of their gestational age at enrolment. The median gestational age at first questionnaire completion was 13 wks. Mothers who were enrolled before gestational age < 18 wks received a postal questionnaire during the second trimester (median gestational age = 20 wks). Mothers who were enrolled before gestational age < 18 and between 18 and 25 weeks of gestation further received a third trimester postal questionnaire (median gestational age = 30 wks). Data from all three questionnaires were used to identify the maternal smoking status during pregnancy. Mothers who reported in the first questionnaire that they smoked during the first trimester only (n=921), but still reported smoking in the second or third trimester questionnaire (n=312) were reclassified into the ‘continued smoking during pregnancy’ category. The same strategy was used for women who reported no smoking in the first questionnaire, but reported smoking in the second or third questionnaire (n=80). The number of cigarettes smoked among those reporting smoking was assessed in six categories at each time point: < 1, 1-2, 3-4, 5-9, 10-19, and ≥ 20 cigarettes per day. For analysis in continued smokers, the smoking quantity reported in all three questionnaires was averaged. Information on partner’s smoking was assessed in the first questionnaire by asking mothers whether their partners smoked during pregnancy including the number of cigarettes smoked per day (1, 1-2, 3-4, 5-9, 10-19, and ≥ 20 cigarettes per day). These questions were also completed by the partners themselves at recruitment in a subset of the cohort (n = 3,558) and agreement in response between partner’s self-report and maternal report was good (sensitivity: 91%; specificity: 95%) [2]. For analysis, we used partner smoking measures from maternal report, as these measures were available for a larger number of singletons and have previously been reported to be a reliable proxy for assessing partner’s smoking effects.

Mothers and their partners had their height and weight measured at enrolment (at ~ 13 wks) in light clothing without shoes, from which early pregnancy body mass index was calculated. Information on other maternal covariates (age, parity, education and alcohol intake) was obtained through the baseline questionnaire. Covariate data of mother’s partners were retrieved indirectly from maternal report (i.e. partner’s age and alcohol intake) or the questionnaire completed by the partner himself at enrolment (i.e. partner’s education). For analysis, self-reported educational level was grouped into three categories of ‘low’, ‘intermediate’ and ‘high’ education referring to ‘primary school’, ‘secondary school’ and ‘higher education’ respectively.

In total, maternal smoking and fetal ultrasound data were available for 4682 White European singletons including 3604 (77%) with maternal genotype data and 4206 (90%) with partner smoking data. All participants gave written informed consent and the study was approved by the local medical ethical committee (MEC 198.782/2001/31).

***Born in Bradford (BiB)***

The Born in Bradford study (BiB) is a population-based prospective pregnancy cohort study that enrolled 12,450 pregnant women residing in Bradford (response rate > 80%), a city in the North of England [3]. Women were mainly recruited at their oral glucose tolerance test (OGTT) appointment scheduled at 26-28 wks’ gestation. The cohort is broadly representative of the obstetric population of Bradford in which most births are to mothers of White European or South Asian origin. Partners of women who were recruited into the study were also invited to participate. The study is characterized by detailed phenotyping of both offspring and their parents, through questionnaires, biological sampling, physical examinations and linkage to obstetric medical records. First and second trimester fetal ultrasound scans routinely collected as part of the NHS screening program and birth anthropometric data were accessed through obstetric records. Besides routine ultrasound scan data, third trimester research scans were collected in a random sample of the cohort. Of all participants included in the present study, 855 (22%) had a third trimester scan.

Information on maternal smoking before and during pregnancy was obtained through a single interviewer-administered questionnaire at enrolment (median gestational age = 26 wks). Participants were asked whether they had ever smoked and how much they smoked in the three months prior to pregnancy, during the first trimester and from the fourth month of pregnancy. The number of cigarettes smoked in each of these periods was assessed in four categories (1-5, 6-10, 11-20, and > 20 cigarettes per day). As for GenR, mothers who reported no smoking prior to pregnancy and/or during the first trimester but reported smoking at any time point after the first trimester were reclassified as pre-pregnancy smokers continuing smoking through pregnancy (n = 57). For analysis in continued smokers, the smoking quantity reported during the first trimester and from the fourth month was averaged. Partners of the women who were recruited to the study were also invited to participate, but since most partners did not attend the OGTT appointment and additional attempts of recruitment at follow-up during pregnancy and soon after birth were unsuccessful, only a small number of partners were enrolled in the study. However, the response rate among partners approached was high (> 90 %) and partner’s self-report of smoking during pregnancy was used for analysis. Quantity of cigarettes smoked was categorized as for the mothers (1-5, 6-10, 11-20, and > 20 cigarettes per day).

Weight and height measurements of the mother and partner were performed in light clothing without shoes. Maternal early pregnancy BMI was calculated based on mother’s weight measured at the first antenatal visit at a median gestational age of 12 wks (as recorded in obstetric medical records) and height measured at recruitment (~ 26-28 wks’ gestation). Mother’s partners BMI was calculated based on the partner’s height and weight measured at enrolment. Information on other maternal covariates was obtained through the questionnaire administered at recruitment (age, alcohol intake and education) or obstetric medical records (parity). Covariate data of mother’s partners was retrieved directly from partner’s self- report at enrolment. Highest educational qualifications were equalized into categories using UK NARIC (<http://www.ecctis.co.uk/naric/default.aspx>) and for analysis collapsed into three groups (‘low’, ‘intermediate’ and ‘high’) representing < 5 GCSEs equivalent, 5 GCSEs equivalent and ≥ A level equivalent respectively.

In total, maternal smoking and fetal ultrasound data were available for 3939 White European singletons including 2923 (74%) with maternal genotype data and 1331 (34%) with partner smoking data. All participants provided written informed consent and ethical approval for the study was granted by the Bradford National Health Service Research Ethics Committee (ref 06/Q1202/48).

**Genotyping and quality control**

We selected the rs1051730 single nuclear polymorphism (SNP) as an instrumental variable for our MR analyses. In GenR, the maternal SNP was typed by LCG (<https://www.lgcgroup.com/genotyping/#.WjD54VVl-po>) using the Taqman allelic discrimination assay (Applied Biosystems, Foster City, CA, USA) and the fetal SNP was extracted from genome-wide data undertaken in fetal cord blood using Illumina HumanHap 610 or 660 Quad chips.

In BiB this SNP was extracted from genome-wide data that had been obtained on maternal pregnancy and fetal cord blood samples using the Illumina HumanCoreExome chip. Details of quality control measures for genotyping are summarized in **S1 Table**. Allele frequencies were similar in both studies and were also similar to previous reports in European populations (frequencies did not deviate from Hardy-Weinberg equilibrium).

**Statistical methods**

***Analysis of fetal growth trajectories with model specification***

Descriptive statistics of the repeat ultrasound and birth anthropometric measurements in the GenR and BiB cohort are summarized in **S2 Table**. In both cohorts, the median number of HC, FL and weight measures per singleton was 3, with the range of measurements being larger in BiB. This is because BiB, by design, includes measures from all routinely collected ultrasound scans. Because AC was not assessed at birth in GenR, the median number of AC measurements per singleton was lower in this cohort (2). Overall, the mean gestational age at anthropometric measurement was somewhat higher in BiB than GenR, which can be explained by the larger number of repeat ultrasound and birth anthropometric measurements in BiB.

We used multilevel models for repeat measurement data to estimate the growth trajectory of each fetal parameter (head circumference (HC), femur length (FL), abdominal circumference (AC) and estimated fetal weight (EFW)). These multilevel models comprise of two levels: measurement occasion (level 1) clustered within the individual (level 2). Fetal ultrasound and birth measurements were approximately normally distributed at different gestational ages and were entered untransformed in analyses. We used fractional polynomial curves with two powers of gestational age from a set of powers (-2, -1, -0.5, 0, 0.5, 1, 2, 3) to identify the best-fitting trajectory for each fetal growth parameter. The best fitting growth trajectory in both cohorts was identified by the following set of powers: HC (powers 1 and 3), FL (powers 1 and 2), AC (powers 2 and 2) and EFW (powers 2 and 3) which were entered as fixed and random effects at the individual level (level 2) of the model, allowing each individual to have its own growth trajectory. For HC and FL, we were able to estimate trajectories from 12 weeks to birth and for AC and EFW from 16 weeks to birth; there were too little data prior to these time points for precise estimation. Model fit was judged by comparing predicted versus observed measurements during different time periods of gestation by checking the distribution of individual level residuals. The predicted measurements of all fetal growth models were broadly consistent with those observed (**S5 Table**). The multilevel fractional polynomial model for each fetal parameter is specified below:

Head circumference (HC):

*y*_ij_ = *β*_0_ + *µ*_0j_ + (*β*_1_ + *µ*_1j_)*t_ij_* + (*β*_2_ + *µ*_2j_)*t_ij_^3^* + *e*_ij_

Femur length (FL):

*y*_ij_ = *β*_0_ + *µ*_0j_ + (*β*_1_ + *µ*_1j_)*t_ij_* + (*β*_2_ + *µ*_2j_)*t_ij_^2^* + *e*_ij_

Abdominal circumference (AC):

*y*_ij_ = *β*_0_ + *µ*_0j_ + (*β*_1_ + *µ*_1j_)*t_ij_^2^* + (*β*_2_ + *µ*_2j_)*t_ij_^2^*log*t_ij_* + *e*_ij_

Estimated fetal weight (EFW):

*y*_ij_ = *β*_0_ + *µ*_0j_ + (*β*_1_ + *µ*_1j_)*t_ij_^2^* + (*β*_2_ + *µ*_2j_)*t_ij_^3^* + *e*_ij_

where *y*_ij_ is the value of HC, FL, AC or EFW at gestational age (*t_ij_)* for individual *j*. Gestational age was set at 12 weeks (HC, FL) or 16 weeks (AC, EFW) with *β_0 ,_ β_1_* _and_ *β_2_* representing respectively the average intercept and coefficients for each power term of gestational age. As two identical variables cannot be entered into a statistical model, we could not enter gestational age to the power of 2 twice in the AC model. Therefore, the second power term for AC was multiplied by the log of gestational age; the data fitted the model well with this adaptation (**S3 Table** and **S2 Fig**). Deviations from the average intercept and power term coefficients for individual *j* are represented by *µ*_0j_, *µ*_1j_ and *µ*_2j_ respectively. The *e*_ij_ term describes the deviation of the *i^th^* measurement on the *j^th^* individual from the individual growth curve. This is the residual error term. For HC, AC and EFW, the residual error was allowed to vary over time (to account for differences in measurement error between ultrasound and birth measurements and increase in measurement variance over time) by defining *e*_ij_ as a function of gestational age. The increase in variance over time was largest for AC and EFW, reflecting the difficulty of AC measurement towards the end of pregnancy because of the ‘hunched’ posture of the fetus (see **S3 Table**). In all models, off-diagonal parameters of the level 1 variance/covariance matrix were set to zero.

To account for potential between-study differences, all analyses models were standard adjusted for study. We also ran all analyses separately within each cohort and tested for between study-heterogeneity by adding a multiplicative interaction term (of study with each of the smoking exposure variables) and its corresponding interaction with gestational age to the models. The following variables were included as potential confounders in multivariable parental smoking analyses: infant sex (male, female) age (continuous in years), parity (at the time of index pregnancy (primiparous, 1, 2, ≥ 3); mothers only), height (continuous in m), body mass index (continuous in kg/m^2^), education (low, moderate, high), and alcohol intake during pregnancy (no, yes). In the parental negative control comparison, associations were additionally mutually adjusted for the smoking behaviour of the other parent: smoking during pregnancy (maternal: non-smokers, pre-pregnancy smokers who quit in early pregnancy, pre-pregnancy smokers who continued through pregnancy; and partner: smokers, non-smokers) and smoking quantity (maternal and partner: none, light, moderate, heavy).

Parental smoking variables and potential confounding variables were included as main effect and as interactions with each of the power terms of gestational age in all models. Global p values for these coefficients were derived in order to assess overall differences in fetal growth. Apart from evaluating overall differences in growth, we also estimated differences in mean fetal HC, FL, AC and EFW at 4-weekly intervals from 12 or 16 weeks onwards. Mean differences (Δ mean) in fetal size by each smoking exposure were reported in absolute original units (i.e. mm and grams) and also proportionally as the ratio of the observed mean difference to the mean (Δ mean / mean ratio) at each time point, as we expect absolute differences to increase with increasing gestational age as fetal size increases.

***Handling of missing covariate data***

The number of singleton pregnancies with missing data on at least one of the potential confounders included in maternal smoking analyses were 989 (21.1%) in GenR and 673 (17.1%) in BiB. The percentage of singletons with missing confounder data in partner smoking analyses was 35.3% and 29.2% in GenR and BiB respectively. Missing covariate data were imputed under the ‘missing at random assumption’ using multivariate multiple imputation with chained equations stratified by cohort, and 10 imputed datasets were generated. The imputation models were specified for each fetal growth parameter (HC, FL, AC and EFW) and included up to 3 repeat measures of fetal size (< 20 weeks’ gestation, 20-40 weeks’ gestation and birth) with corresponding gestational ages, maternal and partner smoking exposures and all covariates included in any of the analysis models.

**References**

1. Jaddoe VW, Mackenbach JP, Moll HA, Steegers EA, Tiemeier H, Verhulst FC, et al. The Generation R Study: Design and cohort profile. Eur J Epidemiol. 2006;21: 475-484.

2. Taal HR, de Jonge LL, van Osch-Gevers L, Steegers EA, Hofman A, Helbing WA, et al. Parental smoking during pregnancy and cardiovascular structures and function in childhood: the Generation R Study. Int J Epidemiol. 2013;42: 1371-1380.

3. Wright J, Small N, Raynor P, Tuffnell D, Bhopal R, Cameron N, et al. Cohort Profile: the Born in Bradford multi-ethnic family cohort study. Int J Epidemiol. 2013;42: 978-991.
