# Supplementary material for: Associations of maternal quitting, reducing, and continuing smoking during pregnancy with longitudinal fetal growth: Findings from Mendelian randomization and parental negative control studies
Source: PLoS Med. 2019 Nov 13;16(11):e1002972. doi: 10.1371/journal.pmed.1002972 (PMC6853297; doi:10.1371/journal.pmed.1002972)
Supplement: S2 Text — (DOCX) [file pmed.1002972.s032.docx]

**S2 Text. Analysis plan.**

Written by: Judith S Brand, Kate Tilling and Deborah A Lawlor

Study population

This project aims to use maternal, paternal and offspring data from the Born in Bradford (BiB) and Generation R (Gen-R) studies, two population-based prospective pregnancy cohorts including participants from multi-ethnic urban populations. For the present analysis, we will include singleton pregnancies without known fetal/birth congenital anomalies. We will further restrict the analysis cohort to participants of White European origin, as smoking behaviours and fetal growth trajectories differ considerably by ethnicity, with, for example, too few of the Pakistani women in the BiB cohort smoking to allow meaningful analyses, and because for some of the ethnic groups in either cohort the number of participants are too small for separate analyses.

Exposures

Based on available maternal questionnaire data, smoking status before and during pregnancy will be defined as (1) *non-smokers* (i.e. women who had never smoked or stopped smoking several months (> 6 months in GenR and > 3 months in BiB) prior to pregnancy); (2) *pre-pregnancy smokers who quit in early pregnancy* (i.e. women who reported smoking in the months before becoming pregnant, in the first trimester but not after the first trimester) (3) *pre-pregnancy smokers who continued through pregnancy* (i.e. women who reported smoking in the months before becoming pregnant, in the first trimester and during mid and/or late pregnancy). Data on smoking quantity will be harmonized by allocating women who continued smoking during pregnancy to categories of ‘light’ (BiB: 1-5 cigarettes per day; Gen-R: < 1, 1-2, 3-4 cigarettes per day), ‘moderate’ (BiB: 6-10 cigarettes per day: Gen-R: 5-9 cigarettes per day) and ‘heavy’ smokers (BiB: 11-20, > 20 cigarettes per day; Gen-R: 10-19 and ≥ 20 cigarettes per day). We will use maternal rs1051730 genotype as an instrumental variable for our Mendelian Randomization (MR) analyses. Paternal smoking status during pregnancy will be defined as binary variable (yes vs. no around the time of their partners’ pregnancy) and categorical variable (for smoking quantity, same as defined for mothers above).

Outcomes

Longitudinal fetal growth trajectories will be estimated based on repeat fetal ultrasound and birth measures, starting from 12/16 weeks of gestation through to term. The following fetal growth parameters with corresponding gestational ages will be included in the analyses: HC (ultrasound and birth), FL (ultrasound), AC (ultrasound and birth) and EFW (ultrasound estimated using the Hadlock formula and weight measured at birth).

Covariates

We will include the following covariates in multivariable adjusted analyses (including the negative control analyses with maternal and paternal smoking association comparisons): offspring sex, age, parity (maternal only), height, body mass index, education level and alcohol use during (or around the time of partners) pregnancy.

Statistical analyses

Analyses with repeat ultrasound and birth anthropometric measurements will be undertaken using MLwiN run in Stata. We will estimate fetal growth trajectories by applying multilevel models with two levels [for measurement occasion (level 1) and the individual (level 2)] with adjustment for cohort (BiB vs. Gen-R). By pooling the data in a single analysis model, we assume that both cohorts are from the same underlying population to which inferences can be made. To test this assumption, we will also use a two-stage analysis model in which we allow for between-study heterogeneity in individual growth patterns and associations with the exposure variables. We will use fractional polynomial curves with two powers of gestational age from a set of powers (-2, -1, -0.5, 0, 0.5, 1, 2, 3) to identify the best-fitting trajectory of each fetal growth parameter in BiB and Gen-R. Differences in fetal growth by maternal smoking status, smoking quantity, rs1051730 genotype and paternal smoking during pregnancy will be analyzed by adding these variables and their interactions with gestational age to the multilevel models. Differences in mean HC, FL, AC and EFW will be estimated at 2-weekly intervals to evaluate the timing at which deviations in fetal size emerge. Associations with maternal smoking status and smoking quantity will be presented adjusted for cohort only and with additional adjustment for offspring sex, age, parity (maternal only), height, body mass index, education level and alcohol use during pregnancy. The same analysis approach will be used for assessing associations with paternal smoking during pregnancy. In the parental negative control comparison, we will additionally adjust associations for the smoking behaviour of the other parent

Associations with maternal rs1051730 genotype will be modelled per risk allele increase and stratified by smoking status to test for horizontal pleiotropic effects of the variant. Covariables will not be included in these analyses, as our underlying assumption is that genetic variants are not widely associated with confounders, but we will explore evidence for this in both studies by examining the association of rs1051730 with potential maternal confounders (see above). To address the possibility of effects of rs1051730 being mediated through inheritance instead of maternal intrauterine effects, we will also perform a sensitivity analysis with additional adjustment for offspring rs1051730 genotype.

In both cohorts, missing covariate data will be imputed using multivariable multiple imputation (10 imputation sets). The imputation model will be specified for each cohort and each individual fetal parameter (HC, AC, FL and EFW) including up to 3 repeat measures of the parameter and corresponding gestational age, the exposure, all covariates and additional variables which are likely to explain some of the missing data. We will also present results from complete case analyses.
