# Supplementary material for: Associations of maternal quitting, reducing, and continuing smoking during pregnancy with longitudinal fetal growth: Findings from Mendelian randomization and parental negative control studies
Source: PLoS Med. 2019 Nov 13;16(11):e1002972. doi: 10.1371/journal.pmed.1002972 (PMC6853297; doi:10.1371/journal.pmed.1002972)
Supplement: S3 Text — (DOCX) [file pmed.1002972.s033.docx]

**S3 Text. STROBE Checklist.**

STROBE Statement—Checklist of items that should be included in reports of ***cohort studies***

|  | Item No | Recommendation |  | Section and paragraph |  |
| --- | --- | --- | --- | --- | --- |
| **Title and abstract** | 1 | (*a*) Indicate the study’s design with a commonly used term in the title or the abstract |  | Title;  Abstract, Methods and findings |  |
|  |  | (*b*) Provide in the abstract an informative and balanced summary of what was done and what was found |  | Abstract, Methods and findings |  |
| Introduction | | |  |  |  |
| Background/rationale | 2 | Explain the scientific background and rationale for the investigation being reported |  | Introduction, Paragraph 1 and 2 |  |
| Objectives | 3 | State specific objectives, including any prespecified hypotheses |  | Introduction, Paragraph 2, Paragraph 3 |  |
| Methods | | |  |  |  |
| Study design | 4 | Present key elements of study design early in the paper |  | Methods, Cohorts |  |
| Setting | 5 | Describe the setting, locations, and relevant dates, including periods of recruitment, exposure, follow-up, and data collection |  | Methods, Cohorts |  |
| Participants | 6 | (*a*) Give the eligibility criteria, and the sources and methods of selection of participants. Describe methods of follow-up |  | Methods, Cohorts |  |
|  |  | (*b*) For matched studies, give matching criteria and number of exposed and unexposed |  | NA |  |
| Variables | 7 | Clearly define all outcomes, exposures, predictors, potential confounders, and effect modifiers. Give diagnostic criteria, if applicable |  | Methods, Parental smoking, Instrument selection and genotyping for Mendelian randomization, Fetal growth assessment, Other variables |  |
| Data sources/ measurement | 8* | For each variable of interest, give sources of data and details of methods of assessment (measurement). Describe comparability of assessment methods if there is more than one group |  | Methods, Parental smoking, Instrument selection and genotyping for Mendelian randomization, Fetal growth assessment, Other variables; S1 Text, Study cohorts, Genotyping and quality control, Statistical Methods |  |
| Bias | 9 | Describe any efforts to address potential sources of bias |  | Introduction, Paragraph 3; Methods, Statistical analysis; S1 Text, Statistical Methods |  |
| Study size | 10 | Explain how the study size was arrived at |  | Methods, Cohorts; S1 Fig |  |
| Quantitative variables | 11 | Explain how quantitative variables were handled in the analyses. If applicable, describe which groupings were chosen and why |  | Methods, Parental smoking, Other variables, Statistical analysis; S1 Text, Statistical Methods |  |
| Statistical methods | 12 | (*a*) Describe all statistical methods, including those used to control for confounding |  | Methods, Statistical analysis; S1 text, Statistical Methods |  |
|  |  | (*b*) Describe any methods used to examine subgroups and interactions |  | Methods, Statistical analysis; S1 Text, Statistical Methods |  |
|  |  | (*c*) Explain how missing data were addressed |  | Methods, Statistical analysis; S1 text, Handing of missing covariate data |  |
|  |  | (*d*) If applicable, explain how loss to follow-up was addressed |  | NA |  |
|  |  | (*e*) Describe any sensitivity analyses |  | Methods, Statistical analysis |  |
| Results | | |  |  |  |
| Participants | 13* | (a) Report numbers of individuals at each stage of study—eg numbers potentially eligible, examined for eligibility, confirmed eligible, included in the study, completing follow-up, and analysed |  | Methods, cohorts |  |
|  |  | (b) Give reasons for non-participation at each stage |  | Methods, Cohort; S1 Fig |  |
|  |  | (c) Consider use of a flow diagram |  | S1 Fig |  |
| Descriptive data | 14* | (a) Give characteristics of study participants (eg demographic, clinical, social) and information on exposures and potential confounders |  | Results, Study population characteristics, Table 1; S4 Table, S5 Table, S6 Table |  |
|  |  | (b) Indicate number of participants with missing data for each variable of interest |  | Results, Table 1; S4 Table, S5 Table, S6 Table |  |
|  |  | (c) Summarise follow-up time (eg, average and total amount) |  | NA |  |
| Outcome data | 15* | Report numbers of outcome events or summary measures over time |  | S1 Table |  |
| Main results | 16 | (*a*) Give unadjusted estimates and, if applicable, confounder-adjusted estimates and their precision (eg, 95% confidence interval). Make clear which confounders were adjusted for and why they were included |  | Results, Associations of maternal smoking with fetal growth, Association of maternal rs1051730 genotype with smoking and fetal growth – Mendelian randomization analysis, Association of maternal vs. mother’s partner smoking with fetal growth – parental negative control analysis |  |
|  |  | (*b*) Report category boundaries when continuous variables were categorized |  | NA |  |
|  |  | (*c*) If relevant, consider translating estimates of relative risk into absolute risk for a meaningful time period |  | NA |  |
| Other analyses | 17 | Report other analyses done—eg analyses of subgroups and interactions, and sensitivity analyses |  | Results, Additional analyses |  |
| Discussion | | |  |  |  |
| Key results | 18 | Summarise key results with reference to study objectives |  | Discussion, Paragraph 1 |  |
| Limitations | 19 | Discuss limitations of the study, taking into account sources of potential bias or imprecision. Discuss both direction and magnitude of any potential bias |  | Discussion, Strengths and limitations |  |
| Interpretation | 20 | Give a cautious overall interpretation of results considering objectives, limitations, multiplicity of analyses, results from similar studies, and other relevant evidence |  | Discussion, Strengths and limitations, Comparison with other studies |  |
| Generalisability | 21 | Discuss the generalisability (external validity) of the study results |  | Discussion, Strengths and limitations |  |
| Other information | | |  |  |  |
| Funding | 22 | Give the source of funding and the role of the funders for the present study and, if applicable, for the original study on which the present article is based |  | Acknowledgments |  |

*Give information separately for exposed and unexposed groups.

**Note:** An Explanation and Elaboration article discusses each checklist item and gives methodological background and published examples of transparent reporting. The STROBE checklist is best used in conjunction with this article (freely available on the Web sites of PLoS Medicine at http://www.plosmedicine.org/, Annals of Internal Medicine at http://www.annals.org/, and Epidemiology at http://www.epidem.com/). Information on the STROBE Initiative is available at http://www.strobe-statement.org.
